# Interventions facilitating access to perinatal care for migrant women without medical insurance: a scoping review

**DOI:** 10.1371/journal.pone.0357487

**Published:** 2026-09-10

**Authors:** Drissa Sia, Idrissa Beogo, Gisèle Mandiangu Ntanda, Eric Tchouaket Nguemeleu, Nebila Jean-Claude Bationo, Catherine Séguin, Geneviève Roch, Christina Greenaway

**Affiliations:** 1 Department of Nursing, Université du Québec en Outaouais, Saint-Jérôme, Québec, Canada; 2 Department of Nursing, Université d’Ottawa, Ottawa, Canada; 3 Library, Université du Québec en Outaouais, Saint-Jérôme, Quebec, Canada; 4 Faculty of Nursing, Université Laval, Quebec, Canada; 5 Jewish General Hospital and Department of Medicine, McGill University, Montreal, Quebec, Canada; University of Greenwich, UNITED KINGDOM OF GREAT BRITAIN AND NORTHERN IRELAND

## Abstract

**Introduction:**

Poor antenatal monitoring is associated with higher rates of maternal complications at delivery and serious threats to neonatal well-being. This scoping review intended to explore existing interventions and policies addressing access to perinatal care among pregnant migrant women without medical insurance (PMWMI). Additionally, it will highlight the strengths and weaknesses of these interventions, as well as their associated costs.

**Materials and Methods:**

Using the Arksey and O'Malley (2005) framework – selected for its structured and widely used approach to mapping the breadth and nature of available evidence -, an electronic search was conducted across 11 databases for studies published between 2000 and 2025 in English or French. Grey literature was also examined. A total of 14 studies were included: 4 from Canada, 8 from the United States, 1 from Iran, and 1 from Thailand.

**Results:**

The gaps created by policy changes, such as the reduction of services, create a misleading perception of economic efficiency. While short-term savings may be evident, they often lead to greater expenses in the medium to long term. In a federal system of government, the financial burden is shifted to the states (or provinces), which are compelled to address the resulting shortfall. The findings indicate that these changes appear to be politically motivated rather than economically beneficial.

**Conclusion:**

This scoping review highlighted interventions that facilitate access to perinatal care for migrant women without medical insurance. As the first of its kind, the findings reveal significant policy shifts that jeopardize the health benefits of undocumented immigrants and asylum seekers, contributing to profound health disparities. Further, these individuals may forgo necessary medical treatment, preventive care, and routine check-ups due to fear of detection and deportation.

## Introduction

Across high‑income countries, uninsured migrant women face substantial barriers to accessing adequate prenatal care [1]. In Toronto, Canada, for example, 80% of uninsured pregnant women received inadequate prenatal care, with more than half receiving clearly inadequate care and 6.5% receiving none at all [2]. Insurance status has been linked to differences in the type of healthcare provider seen, indications for caesarean section, neonatal resuscitation rates, and the length of maternal hospital stay among uninsured migrant, refugee, asylum‑seeking, and undocumented women [2–5]. Similar patterns have been documented internationally, where immigrant women frequently encounter inadequate prenatal care [6–9], contributing to higher stillbirth rates [10]. Those lacking medical insurance often initiate care late in pregnancy and facing more severe delivery complications [11], including more emergency caesarean sections, which can result in lasting trauma for both mother and child [11]. In both Canada and the United States, many migrant women receive care that falls below recommended standards because they are excluded from public health insurance coverage [2].

This review focus on migrant women aged 12 years and older who lack health insurance and are either pregnant, in labor, or postpartum. According to the glossary of the International Organization for Migration, the term “migrant” refers to “a person who is compelled to move, whether within a country or across an international border, temporarily or permanently, for a variety of reasons, to improve his or her material and social conditions, as well as those of his or her family [12].

In Canada, refugee claimants have coverage through the Interim Federal Health Program (IFHP) for medical and hospital services that is virtually identical to those afforded citizens, as well as full coverage of prescription medications and partial coverage of other services, including midwives. However, this is not always observed in practice in other contexts, as highlighted in the literature [13, 14]. Certain categories of migrants with temporary visas, as well as undocumented migrants, receive no health coverage at all [15]. Migrants are also disproportionately affected by financial vulnerability [16]. In many countries, restrictive immigration policies, such as mandatory waiting periods for public insurance, exclusion of temporary residents from health coverage, and the denial of services to undocumented individuals, further limit access to essential maternal healthcare [17–19]. As a result, migrant women frequently face inadequate prenatal care [9, 20], which contributes to higher rates of stillbirth, fetal growth restriction, preterm birth, and disproportionate perinatal mortality [21–23].

Migrants without medical insurance are particularly vulnerable populations [10, 11]. They often begin their prenatal consultations late in pregnancy [1] what leads to medical complications during labor [11]. Additionally, they have a higher incidence of emergency caesarean sections, contended to be traumatic for both the mother and the future child [10]. One systematic review and meta-analysis, comparing pregnant women with an immigration background to native origin, showed that the former had significantly higher risks of emergency caesarean section (OR = 1.1, 95% CI = 1.0–1.2), shoulder dystocia (OR = 1.1, 95% CI = 1.0–1.3), gestational diabetes mellitus (OR = 1.4, 95% CI = 1.2–1.6), small for gestational age (OR = 1.3, 95% CI = 1.1–1.4), low 5-min Apgar score (<7) (OR = 1.2, 95% CI = 1.0–1.3), and oligohydramnios (OR = 1.8, 95% CI = 1.0–3.3) [24].

A substantial burden of postpartum depression [25], parasitic infections [26], and other communicable diseases (e.g., hepatitis B, hepatitis C, and HIV/AIDS) [27] has also been reported among this population. The combination of their precarious status and the challenges they face in accessing perinatal care [28] multiplies the burden for them. The matter of pregnant migrant women without medical insurance (PMWMI) is complex. To improve perinatal care, interventions have focused on providing early and routine services, delivering culturally sensitive programs, enhancing geographic accessibility, fostering multidisciplinary collaboration, and integrating care with affordable community resources [29, 30]. Previous reviews have examined migrant maternal health more broadly [31] or focused on outcomes among asylum seekers and undocumented migrants [32, 33], but none have specifically synthesized interventions aimed at improving access to perinatal care for migrant women without medical insurance or with precarious migration status.

No knowledge synthesis has yet examined interventions aimed at improving access to perinatal care for PMWMIs. Given the growing concerns surrounding healthcare access for PMWMIs, conducting a comprehensive review to catalogue existing interventions that facilitate perinatal care is both timely and essential. That is, we run a scoping review of interventions and policies to synthesize the available evidence, assess their strengths and weaknesses, and evaluate their financial costs. This understanding will facilitate the implementation of context-appropriate interventions to improve access to perinatal care for PMWMIs.

Interventions comprised a comprehensive package of maternal and newborn health services, including pregnancy testing, prenatal care and follow-up, and screening for diseases during pregnancy. It provided immediate obstetric and neonatal support during labor, delivery, and the first two hours postpartum, as well as postpartum and newborn care. Additional components included prenatal education, vaccination, and assistance during childbirth, ensuring continuity of care across the maternal and neonatal period. Policy-level interventions were incorporated to support and sustain these services. Additionally, knowledge of the costs associated with these interventions will assist decision-makers in recognizing the financial benefits of investing in their establishment. This scoping review addresses a gap in the literature and aims to identify interventions that have been utilized to enhance access to perinatal care for PMWMIs. It also emphasizes the strengths, weaknesses, and costs of these interventions.

### Research questions

This review examines interventions and policies that enhance PMWMIs’ access to perinatal care and addresses two key research questions.

1. What are the interventions that facilitate access to perinatal care for PMWMI women?
2. What are the interventions impacts on PMWMI perinatal health
3. What are the strengths and weaknesses of the interventions, and what is the cost of implementation?

## Materials and Methods

### Methodological framework

The protocol of this review has been published [34]. This study utilized the Arksey and O’Malley framework [35]. It provides six fundamental steps for carrying out a thorough scoping review: (i) defining the research question; (ii) identifying relevant studies; (iii) selecting studies; (iv) extracting data; (v) analyzing and aggregating results; and (vi) conducting a consultation exercise (optional). As the consultation phase is optional, it was not undertaken in this review.

### Data sources and research strategy

The study was registered in Research Registry (#6864 https://www.researchregistry.com/browse-the-registry/#home/?view_2_search=6864&view_2_page=1) and followed PRISMA-ScR recommendations [36] (see S1 Checklist). Eleven electronic databases for studies published between 2000 and 2024 were queried, including CINHAL, Web of Science, Medline-Ovid, Embase, Cochrane Library, Scopus, ScienceDirect, Hinari, Lilacs, Cairn and Banque de Données Santé Publique (BDSP). Additionally, we performed search in other sources including 1) reference tracking from reference lists; 2) key journals in the field of immigration (Revue Migrations Forcées; Migrations Société); and 3) Grey literature, including websites of non-governmental organizations (NGOs) such as Médecins du Monde, Médecins Sans Frontières (MSF), and the United Nations High Commissioner for Refugees (UNHCR).

A working meeting with the research team, which included an experienced librarian (CS), facilitated the development of the search strategy (presented in Table 3 published elsewhere [34]. An expanded version, including the complete search strategies for all databases consulted, is presented in Table 3 of the present review. This strategy was formulated using descriptors or thesaurus along with the logical operators “AND” and “OR” to identify relevant studies published in French or English. An updated screening was conducted in December 2024. The year 2000 was chosen as a reference point with the launch of eight Millennium Development Goals (MDGs), addressing gender equality (MDG 3), and child mortality reduction (MDG 4) [37].

### Study selection

#### Inclusion Criteria.

Inclusion and exclusion criteria were based on the Population, Interventions, Comparators, and Design and Outcomes, or Anticipated Outcomes (PICO), as summarized in Table 1 in the published protocol [34].

**Table 1:** Summary of studies included in the review (n = 14).

Study characteristics		
**Year of publication**	Studies	N
2000-2005	Lu et al. (2000)	1
2006-2010	Fuentes-Afflick et al. (2006); Rodriguez et al. (2010)	2
2011-2015	Crozier et al., (2013); Jarvis et al. (2011); Wilson-Mitchell and Rummens (2013)	3
2016-2020	Atkins et al. (2018); Darling et al. (2019); Médecins du monde (2020); Mohammadi et al. (2017); Rodriguez et al. (2020); Swartz et al. (2017); Wherry et al. (2017)	7
2021-2025	Kim et al. (2025)	1
**Country of the study**		
Canada	Darling et al. (2019); Jarvis et al. (2011); Médecins du monde 2020;; Wilson-Mitchell and Rummens (2013)	4
Iran	Mohammadi et al. (2017)	1
United States of America	Atkins et al. (2018); Lu et al. (2000); Fuentes-Afflick et al. (2006); Swartz et al. (2017); Rodriguez et al. (2010); Rodriguez et al. (2020); Wherry et al. (2017); Kim et al. (2025)	7
Thailand	Crozier et al., (2013)	1
**Study design**		
Cross-sectional descriptive study (Audit study method)	Fuentes-Afflick et al. (2006)	1
Cohort (longitudinal) Study	Atkins et al. (2018); Darling et al. (2019); Swartz et al. (2017); Lu et al. (2000); Rodriguez et al. (2010); Wilson-Mitchell and Rummens (2013)	6
Qualitative study (semi-structured interview)	Mohammadi et al. (2017)	2
Decision analytic model	Rodriguez et al. (2020)	1
Quasi-experimental study	Jarvis et al. (2011); Wherry et al. (2017)	2
Grounded theory study: qualitative design	Crozier et al., (2013)	1
Quantitative retrospective Cross-sectional population-based	Kim et al. (2025)	1
**Sample size**		
< 100	Crozier et al., (2013); Mohammadi et al. (2017)	2
100-999	Jarvis et al. (2011); Lu et al. (2000); Wilson-Mitchell and Rummens (2013)	3
1000-1999	Médecins du monde (2020); Rodriguez et al. (2010); Wherry et al. (2017)	3
≥ 2000	Atkins et al. (2018); Darling et al. (2019); Fuentes-Afflick et al. (2006); Rodriguez et al. (2020); Swartz et al. (2017); Kim et al. (2025)	5

#### Population (P).

Articles addressing the perinatal period of uninsured migrant women were also considered. It is important to note that migrants are not a homogeneous population [12]. Migrants originate from diverse countries and cultures and fall roughly into two groups: (i) those in a regular situation, whose entry and stay in the host country comply with applicable laws; and (ii) those in not regular situation vis-à-vis of the regulations of the host country by the way they get in or because they stay beyond the validity of their residence permit [12, 38]. This review also considers internally displaced persons, as well as refugees and asylum seekers without health insurance.

#### Interventions (I).

This review examines care for uninsured migrants during the perinatal period and the policies that facilitate access. For the purposes of this review, the perinatal period is defined as extending from the onset of pregnancy to the early months of life, thereby expanding beyond the World Health Organization definition [39]. It encompasses pregnancy testing; prenatal care (antenatal follow-up); immediate obstetric and neonatal care (during labor, delivery, and within two hours postpartum); postpartum care (up to 42 days after delivery); and newborn care (up to 28 days of life) [40]. Care, including prenatal education, labor, delivery, and postpartum services, was considered per the MdM framework [41] [summarized in Table 2 of [34]]. Were excluded, intervention falling out of the perinatal period.

#### Comparator and design (C).

As for the design, we considered all empirical scientific studies, of any design, conducted in French or English from any country [42]. This inclusive approach was chosen to capture the full range of interventions and policies implemented to support uninsured migrant women during the perinatal period, acknowledging that the evidence base is limited and methodologically diverse. Media articles, editorial comments, and studies focusing solely on the profiles of PMWMIs were excluded because they do not provide empirical data on interventions or policy measures relevant to our research question. No comparison was anticipated.

#### Outcomes or intended results (O).

Studies were included if they reported the impacts, strengths, limitations, and implementation costs of interventions aimed at improving perinatal care access for PMWMIs.

### Studies Screening

**Fig 1:**
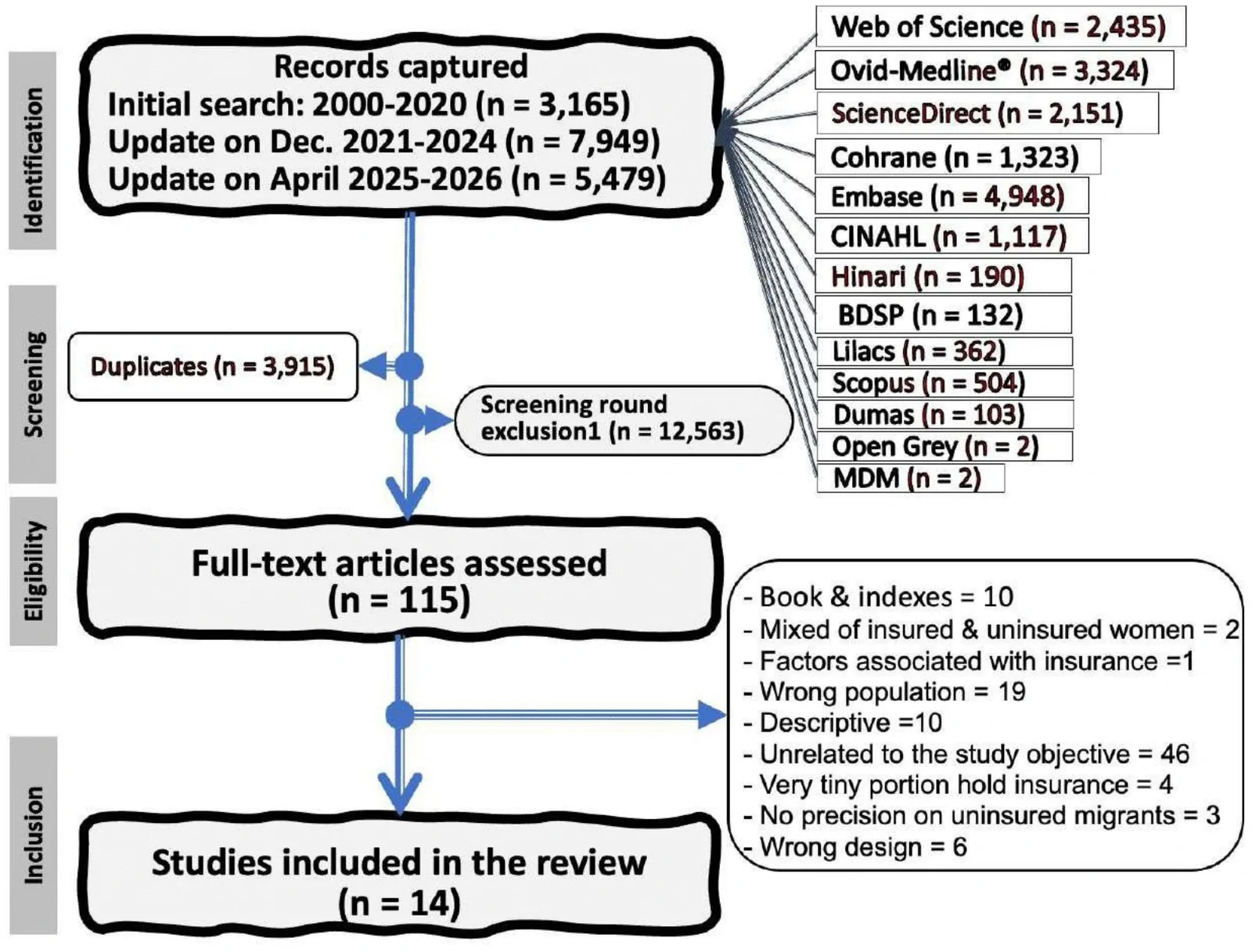
Prisma flow diagram illustrating the search strategy.

In collaboration with the librarian (CS) of the Université du Québec en Outaouais and co-authors (I.B. and D.S.) designed the selection of articles and their extraction strategy. To begin selection, data were exported to EndNote for electronic and manual duplicate removal, then transferred to Rayyan [43]. Article selection proceeded in four steps: first, the team adapted a screening algorithm —develop by the team and used in previous reviews [44–47] — (Fig 1 of the protocol [34]); second, 10% of articles were jointly reviewed to harmonize the process (D.S., I.B., N.J.-C.B., E.T., G.M.N.); third, three authors (D.S., I.B., N.J.-C.B.) independently screened titles and abstracts and abstracts, and an article was retained if at least two reviewers deemed it eligible. Across this stage, 2.32% of screening decisions resulted in a conflict, all of which were resolved through discussion after re‑examining the record. This corresponds to an agreement rate of 97.68%, reflecting strong consistency in the application of eligibility criteria. Over the process, disagreements were resolved through discussion after reviewing the title and the abstract; if consensus was not reached, the fourth author (E.T.) made the final decision.

At the final step, eligible articles have been read in their entirety by two independent reviewers (D.S. and I.B.). At the end of this step, all authors met and summarised the reasons for exclusion of articles to prepare the flow diagram of the study.

### Data extraction

The data extraction grid [S2 Appendix of [34]] was adopted from Cameron et al. [48], to capture study details (authors, year, country, title, design, population, sample size, interventions (antenatal, obstetric/neonatal, postpartum, newborn care, and related policies), impact, strengths, weaknesses, and implementation costs. To ensure consistency, three authors (D.S., I.B., N.J.-C.B.) collaboratively extracted data from two of the 13 included articles, after which N.J.-C.B. completed the remaining extractions. All extracted data were validated by D.S. and I.B.

### Data analysis

Data analysis was conducted using a narrative synthesis informed by thematic analysis. Following data extraction, study findings were systematically coded and compared across study objectives, intervention types, and settings. A thematic synthesis was then undertaken to identify recurring patterns and relationships across the included studies, with themes generated through iterative review and discussion among members of the research team.

While themes emerged inductively from the data, the organisation of the Results section was guided by key outcome domains relevant to the review objectives, including intervention impacts, strengths, weaknesses, and costs. This combined analytical approach enabled a structured comparison of interventions and facilitated the identification of effective strategies for improving access to perinatal care among PMWMI women. The synthesis also informed implications for future research, practice, and policy development.

The study was approved by the Research Ethics Committee of the Université du Québec en Outaouais (Project #2021-1490, March 11, 2021). No informed consent was required, as no individual data were collected.

## Results

### Study characteristics

We identified 16,593 records and removed 3,915 duplicates (last update in April 16, 2026). After screening titles and abstracts, an additional 12, 563 records were excluded (see Flowchart). A total of 14 studies met the inclusion criteria and were included in this review (Table 1). These studies were published between 2000 and 2025. The studies were conducted in four countries: the United States (n = 8) [49–56], Canada (n = 4) [2, 21, 57, 58], Thailand (n = 1) [59], and Iran (n = 1) [14]. Study designs varied and included cohort (longitudinal) studies (n = 6) [2, 49, 52, 53, 55, 57], quasi-experimental studies (n = 2) [21, 56], qualitative studies (n = 1) [14], cross-sectional descriptive studies (n = 1) [50], grounded theory studies (n = 1) [59], Retrospective and decision-analytic models (n = 1) [51]. Sample sizes ranged from fewer than 100 participants (n = 2) [14, 59], 100–999 participants (n = 3) [2, 21, 52], 1,000–1,999 participants (n = 3) [56, 58, 60], to 2,000 or more participants (n = 6) [49–51,54,55,57]. Across publication periods, one study was published between 2000–2005 [52], two between 2006–2010 [50, 60], three between 2011–2015 [2, 21, 59], seven between 2016–2020 [14, 49, 54–58], and one in 2025 [51].

The Prisma flow diagram is presented in Fig 1.

The results are presented according to themes identified through inductive thematic synthesis of the included studies. Four overarching themes structure this section: (1) interventions facilitating access to perinatal care for PMWMIs; (2) barriers affecting PMWMIs’ access to perinatal healthcare; (3) strengths and weaknesses of identified interventions; and (4) costs associated with intervention implementation. Within these themes, subthemes address key dimensions of the evidence, including actors involved in implementing perinatal care policies for PMWMIs, types of policy and programmatic interventions, and reported strengths and limitations. This thematic organisation reflects recurring patterns across the included studies and provides a coherent framework for synthesising findings related to access, implementation, and outcomes of perinatal care interventions for PMWMIs.

In relation to the first theme, across the 14 included studies, we identified eight existing interventions and policies designed to improve access to perinatal care for PMWMIs. These included government‑led policies such as the OHIP, the CHIP, the CHIPRA, CAWEM Plus, and state‑level public health insurance expansions, as well as non‑governmental initiatives such as MdM community‑based services. These interventions covered the full perinatal continuum, including prenatal, postpartum, and newborn‑care components such as postpartum contraceptive access and neonatal care packages. Together, these eight interventions constitute the existing policy landscape documented in the included studies.

1 **Interventions in favor of access to perinatal care by pregnant migrant women without medical insurance (PMWMIs)**
  1.1 Actors involved in implementing perinatal care policies for PMWMIs

Of the studies selected, six [49,54–58] highlight two classes of policies promoting access to perinatal healthcare for PMWMIs, from which two types of actors emerge: intervention by government actors [49,51,54–57] and intervention by non-State actors [58]. The State-level interventions included, in Canada, The Ontario Health Insurance Plan (OHIP) of the Ontario government in force since 1994. As exhibited in the Table 2, in the USA, there was the Alternative policies implemented through Medicaid to address the loss of access to care following restrictive reforms, namely, the Children’s Health Insurance Program (CHIP) [54], the Health Insurance Program Reauthorization Act (CHIPRA) [49, 56] and Citizen/Alien Waived Emergent Medical Care (CAWEM Plus) or Emergency Medicaid Plus, expanded in 2008 by Oregon to include all recent and unauthorized immigrant women in the prenatal care offering [55].

**Table 2:** Interventions in favor of access to perinatal care, barriers, Strengths, weaknesses and costs.

Authors/ year/ reference number	Study population	Interventions in favor of access to perinatal care by pregnant migrant women						Barriers to PMWMIs’ access to perinatal health care		Strengths and weaknesses of these interventions		Policy/Intervention Costs
		Actors involved in implementing perinatal care policies for PMWMIs				Policy Interventions for Access to Perinatal Care						
		Policy	Results	Policy	Results	Policy	Results	Barriers	Results	Strengths	Weaknesses	
		Governmental actors		Nongovernmental actors								
Darling & al. (2019) (43)	Unsured migrants	The Ontario Health Insurance Plan (OHIP), since 1994, has included midwifery care, free and comprehensive perinatal services to all pregnant women residing in Ontario	The uninsured received, on average, more home visits than the insured, with 1.90 ± 3.4 and 0.6 ± 1.1 visits respectively; but certain needs remain unmet: medical consultations and screenings do not meet expectations			Prenatal visit	Uninsured clients had more antenatal home visits than insured clients (1.9 vs 0.6 on average) but were less likely to initiate care in the first trimester (66.3% vs 92.8%) and entered midwifery services later in pregnancy (18.4 vs 12.7 weeks)					
Rodriguez & al. (2020) (44)	Unauthorized immigrant women	Children’s Health Insurance Program (CHIP) and the Unborn Child Clause, which allow states to utilize federal funding to cover woman’s pregnancy and delivery-related care, regardless of legal status				Compared the outcomes and cost-effectiveness of two prenatal care policies: federal standard —delivery coverage only— and prenatal care plus delivery coverage (State Children’s Health Insurance Program) among low-income immigrant women.	The State Children’s Health Insurance Programd strategy was cost-effective. This coverage avert an estimated 117 deaths and 34 cases of moderate to severe cerebral palsy in children			The flexibility of the program allowed Oregon State decided to offer postpartum coverage for all women, irrespective of their citizenship status		For every 865 women who receive prenatal care in addition to delivery services, one infant death can be prevented, representing an estimated cost of $328,700 per life saved.
Atkins & al. (2018) (45)	Unauthorized immigrant women	Health Insurance Program Reauthorization Act (CHIPRA), extended federal policy by eliminating the five-year waiting period, thereby granting full access to comprehensive Medicaid or CHIP coverage for pregnant women, regardless of residency status				Expanded Medicaid coverage to unauthorized women for prenatal care in Nebraska, versus in South Carolina, which has never done so.	In Nebraska, during Medicaid expansion period, the likelihood for unauthorized women to receive adequate prenatal care was quite high (OR = 1.28, p < 0.01) compared with those without expanded Medicaid coverage			During Medicaid expansion, unauthorized women residing in Nebraska were 28% more likely to receive adequate prenatal care and had roughly one additional prenatal visit compared to those without expanded coverage		
Wherry & al. (2017) (46)	Unauthorized immigrant women	Health Insurance Program Reauthorization Act (CHIPRA), extended federal policy by eliminating the five-year waiting period, thereby granting full access to comprehensive Medicaid or CHIP coverage for pregnant women, regardless of residency status				State public health insurance expansions on prenatal care utilization, delivery methods, and infant health among pregnant immigrant women.	There was a 0.2 visit increase on average in prenatal consultations for undocumented immigrants overall. For those with less than a high school education, there was a 0.2 visit increase on average in consultations and an increase of 2.7% in adequate prenatal care.			the extension policy increased the use of prenatal consultations for undocumented immigrants by 0.2 visit on average		
Swartz & al. (2017) (47)	Refugee women	Citizen/Alien Waived Emergent Medical Care (CAWEM Plus) or Emergency Medicaid Plus, expanded in 2008 by Oregon to include all recent and unauthorized immigrant women in the prenatal care offering				compared pregnancies covered by Emergency Medicaid, Emergency Medicaid Plus, and Medicaid	Significant improvements following the expansion of Emergency Medicaid Plus (CAWEM Plus; Citizen/Alien Waived Emergent Medical Care) for unauthorized immigrant women and those recently settled: Prenatal visits rose by 7.2, adequate prenatal care by 28%, diabetes screening by 61%, and fetal ultrasounds by 74%. Well-child visits increased slightly (+0.24), as did screenings and vaccinations (+0.04 probability). Infant mortality fell by 1.04 per 1000, and extremely low birth weight (<1000g) by 1.5 per 1000					
Médecins du monde (2020) (48)	Undocumented migrants women			Interventions through a clinic system in Montreal dedicated to PMWMIs with precarious status: to address the shortcomings of the two levels of government; and the direct provision of care and services to these populations.	Enhances access to healthcare for individuals in vulnerable situations: MDM provides them with free medical services. They are often referred by organizations or other entities they have previously consulted	clinic system dedicated to PMWMIs with precarious status	this organization primarily serves individuals with precarious status, with the majority (85%) being immigrants without medical insurance, including 23% who are pregnant women					
Fuentes-Afflick & al. (2006) (49)	Unauthorized immigrant women					the 1996 Personal Responsibility Work Opportunity Reconciliation Act (PRWORA)	Undocumented immigrants were more likely to inadequately use prenatal care —first trimester visit with fewer than 6 prenatal visits or prenatal visit after the first trimester— in all the study settings with an OR ranging from 1.66, (95%CI=0.94-2.92) in New York to 3.55, (95%CI=1.49-8.43) in Florida	opted not to continue providing services to ineligible groups	Florida had a significantly higher rate of inadequate prenatal care (OR=3.55; 95% CI 1.49–8.43), whereas California (OR=1.91; 95% CI 0.75–4.89) and New York (OR=1.66; 95% CI 0.94–2.92), which continued services for ineligible groups, showed better utilization		the PRWORA is more likely to lead to inadequate use of prenatal care by 2–4 times. Some policies have proven difficult to implement	
Rodriguez & al. (2010) (50)	Unsured migrant women					Hospital provision of intrauterine contraceptive device (IUD) postpartum program versus state funding of IUDs postpartum program	A postpartum IUD program does not generate cost benefits for hospitals					Providing postpartum contraception has proven to save public funds while empowering women to plan their families
Wilson-Mitchell and Rummens (2013) (6)	Uninsured immigrant women					Perinatal care	the majority (81.2%) of uninsured pregnant women received inadequate prenatal care, while 6.5% received none at all. Also, 26.3% of uninsured (compared to 35.6% of insured) experienced C-section for abnormal fetal heart					
Lu & al. (2000) (1)	Undocumented migrants women							A cut of public funding on prenatal care for undocumented women in California	About 9.5% of undocumented immigrant women received no prenatal care, and over half consulted late. Women without care had higher rates of preterm birth (34.8% vs 7.5%, p < 0.001) and faced increased risks of preterm birth (RR = 3.8) and low birth weight (RR = 7.4); Cutting prenatal care funding saved $58 million but added $194 million in postnatal costs			Every $1 cut increased postnatal care costs by $4.63. Attempted savings of $58M resulted in $136M–$211M in additional costs.
Mohammadi & al. (2017) (42)	Afghan Refugees							Despite extended inpatient coverage to Iranians and registered migrants, key experiences included discrimination and inadequate care	Participants noted poor communication, delayed complication recognition, and lasting psychosocial impacts		The study underscored a mistreatment of refugees in Iran following the 2015 reform that public health scheme that now covers inpatient care costs for all Iranians and registered migrants.	
Jarvis & al. (2011) (2)	Undocumented migrant women							Uninsured women in Canada face significant challenges	Uninsured pregnant women began prenatal care later than insured women and underwent fewer screenings and provider visits			Access barriers make perinatal care costly for uninsured immigrant women, but funding these services benefits both them and society
Crozier et al., (2012) (41)	Migrant women							Four main barriers to HIV screening among migrant women: poor information provision, access difficulties, migrant status implications, and risk perception	The following quotes illustrate these barriers: i) “The nurse in the counselling room told me that the details about the blood test were in the book [antenatal clinic booklet]. She never told me why I need to take the test” (Participante 4); ii) Nurse 8: “We did it [bloodtest] for every case. I have never asked them whether they accepted or not. We did not have time”			
Kim, Choi & Kim (2025)	- Immigrant pregnant women; - Foreign-born mothers with singleton births; - Publicly insured and uninsured immigrants							ICHIA option allowing states to enroll lawfully present immigrant pregnant women in Medicaid/CHIP without the 5-year waiting period; FCEP/Unborn Child option using CHIP funds for prenatal care regardless of immigration status; State-only funded insurance programs for immigrant pregnant women excluded from federal Medicaid/CHIP eligibility.	All three policies reduced delays in prenatal care initiation. State-only funded programs showed the strongest improvements in early and adequate prenatal care utilization. Publicly insured immigrant women had higher odds of adequate prenatal care and lower odds of inadequate care.	Expands access to prenatal care for vulnerable migrant women; promotes earlier prenatal care initiation; improves adequacy of prenatal care; State-only programs reduce immigration-related fears; FCEP simplifies enrollment procedures.	ICHIA and FCEP policies showed less consistent effects on adequacy of prenatal care; implementation complexity; overlapping policies complicated evaluation; administrative procedures remained burdensome; sustainability of State-only programs depends on state resources and political context.	State-only funded programs likely require substantial state financial investment and administrative coordination. Medicaid/CHIP-related options may reduce direct state costs through federal support but still involve administrative expenses related to enrollment and eligibility verification.

Regarding the policies of non-governmental actors, the study by MdM [58] was retained, as its initiatives address the shortcomings of both levels of government by directly providing care and services to PMWMIs.

1.2 Policy Interventions for Access to Perinatal Care

Several studies have focused their purpose on Policy Interventions for prenatal care services solely. This is the case of study of Atkins et al. [49], Fuentes-Afflick et al. [50] and Darling et al. [57]. The second category of intervention included both prenatal and postpartum/newborn care. On that perspective, Swart et al. [55] for instance compared pregnancies’ package (e.g., Prenatal visits, diabetes screening, fetal ultrasounds) covered by Emergency Medicaid, Emergency Medicaid Plus, and Medicaid, while Rodriguez et al. [60] looked at women's access to contraceptive methods following delivery, specifically using postpartum intrauterine devices. Besides, Wherry et al.’s [56] study examined the impact of state public health insurance expansions on prenatal care utilization, delivery methods, and infant health among pregnant immigrant women. Finally, in addition to above mentioned portrayal as exhibited in Table 2, other studies were concentrated on newborn care and potential perinatal complications (e.g., cerebral palsy and infant mortality) [54] and neonatal resuscitation and admissions to intensive care units [2]. The Kim et al. [51] study estimated effects of immigrant policies on prenatal care use and timing comparing three policy option: Immigrant Children’s Health Improvement Act (ICHIA), From-Conception-to-the-End-of-Pregnancy (FCEP), and the State-only funds option, publicly insured immigrants. Their data (2333,526 mothers and infants: 1280,847 residents in states with the ICHIA, 1227,879 in states with the FCEP, and 341,101 in states with State-only funding) from National Center for Health Statistics for the years 2015–2019.

2 **Barriers to PMWMIs’ access to perinatal health care**

Studies have revealed several barriers, notably those linked to the reduction of health funding that supported undocumented immigrant women in the United States. The policy changes initiated in 1996 created significant obstacles to accessing prenatal care for this population [52]. The qualitative study by Mohammadi et al. [14] shows that Afghan immigrant women (uninsured) experienced delays and disparities in maternal care provided to them compared to Iranian women in Iran. Participants emphasized serious gaps in communication, significant delays in the recognition and management of complications, and profound, enduring psychosocial repercussions. Jarvis et al. [21] noted that uninsured women in Canada initiated their first prenatal care 13.6 weeks later than their insured counterparts. Additionally, they receive fewer routine prenatal screening tests (e.g., blood tests: 93.7% vs. 100%, p = 0.045, ultrasound screenings: 82.5% vs. 98.4%, p = 0.003).

Fuentes-Afflick et al [50] compared the implementation outcomes of PRWORA, found that Florida exhibited a significantly higher rate of inadequate prenatal care utilization (OR = 3.55). In contrast, California (OR = 1.91) and New York (OR = 1.66), which opted to continue providing services to ineligible groups, demonstrated better utilization of prenatal care. Crozier et al. [59] study highlighted ineffective provision of information, internal and external barriers to accessing information, implications of migrant status, and perceptions of risk (Table 2). Finally, Kim et al. [51] laid out barriers related to: immigration-related restrictions, enrollment burden, public charge fears, lack of coverage, delays in eligibility verification, and communication barriers.

3 **Strengths and weaknesses of perinatal care policies and their implementation**

This theme synthesizes evidence on the strengths and weaknesses of policy and programmatic interventions shaping PMWMIs’ access to perinatal care, including enabling policy mechanisms as well as structural and implementation‑related limitations.

### Strengths

Several studies reported that specific policies enabled expanded access to perinatal care for PMWMIs by allowing flexibility at the provincial or state level. For example, studies conducted in the United States described the application of the Unborn Child Clause of the Children’s Health Insurance Program (CHIP) in Oregon, which permitted coverage for postpartum care regardless of citizenship status [54]. The retrospective cross-sectional study by Kim et al. [51], comparing three schemes, further highlighted the relative strengths of each policy option. The ICHIA: expands access to prenatal care, encourages initiation, improves adequacy, reduces immigration fears, simplifies enrollment procedures; the FCEP: Expands eligibility, improves access to prenatal services, reduces delays in care initiation; while the State-Only: Most effective option; increases first-trimester initiation and adequate care, reduces immigration-related fears via non-federal programs. Theire study [51] informed that the State-funded policy is associated with a greater odds of intermediate (OR 1.43), adequate (OR 1.72), and adequate-plus (OR 1.37) care, with lower inadequate care (OR 0.48) -compared to uninsured immigrants-, increased first-trimester initiation (+87.1 pp), and reduced delayed (−43.8 pp) and no care (−67.3 pp).

Other studies reported that the Health Insurance Program Reauthorization Act (CHIPRA) was associated with expanded eligibility and increased coverage for PMWMIs in several states [49, 56], as summarized in Table 2.

### Weaknesses

Other studies documented limitations and adverse effects associated with certain policies and their implementation. Fuentes‑Afflick et al. [50] reported that the implementation of the Personal Responsibility and Work Opportunity Reconciliation Act (PRWORA) was associated with higher odds of inadequate prenatal care utilization among PMWMIs, with reported odds ratios ranging from approximately two‑ to four‑fold across states. In addition, a qualitative study conducted in Iran by Mohammadi et al. [14] reported that Afghan refugee women experienced persistent mistreatment and disparities in maternal care following the 2015 health reform, despite the extension of national public insurance to registered migrants. Reported issues included delayed care, communication gaps, and differential treatment in clinical settings.

4 **Policy/Intervention Costs**

Several studies reported cost‑related outcomes associated with limited access to insurance coverage for PMWMIs. Jarvis et al. [21] documented higher costs related to delayed or incomplete prenatal care among uninsured immigrant women. The study of Kim et al. [51] informed that the State-funded programs involve substantial implementation costs and administrative coordination burdens. Lu et al. [52] reported that reductions in public funding for prenatal care for undocumented migrants in the United States were associated with increased postnatal healthcare expenditures. Specifically, the authors estimated that an initial reduction of approximately $58 million in prenatal care spending was followed by higher postnatal costs, estimated at $136 million and increasing to $211 million over time.

In addition, Rodriguez et al. [54] reported that providing prenatal care in addition to delivery coverage was associated with an average additional cost of $380 per woman. The study further estimated that for every 865 women receiving prenatal care alongside delivery coverage, one infant death could be averted, corresponding to an estimated cost of $328,700 per life saved.

## Discussion

This scoping review aimed to identify interventions and policies that support access to perinatal care for PMWMIs, and to examine their impacts, strengths, weaknesses, and associated costs. The Discussion synthesises these findings in relation to the review objectives. While the Results were organised according to outcome domains (impacts, strengths, weaknesses, and costs), the findings are interpreted here across broader thematic areas, including policy dynamics, economic considerations, implementation challenges, and ethical and public health implications. References drawn from outside the included studies are used to contextualise the findings and are explicitly indicated as such.

Across the included studies, most were conducted in the United States and Canada, highlighting a geographical concentration of evidence. The findings consistently show that existing policies do not fully cover uninsured migrant populations. Several studies report that changes in immigration or insurance status frequently disrupt access to care, particularly in the United States. From a cost perspective, included studies suggest that extending public coverage to PMWMIs is economically viable and may reduce downstream healthcare expenditures. Two main mechanisms facilitating access were identified: formal policy interventions and the complementary role of NGOs, which often fill gaps in public provision. These findings highlight both strengths (e.g., local adaptability and NGO involvement) and weaknesses (e.g., fragmented coverage and instability) in current systems (Table 3).

**Table 3:** Research strategy.

#	Query
**CINAHL**	
1	TI (Immigrant* OR migrant* OR temporary worker* OR ‘migrant worker*’ OR ‘foreign worker’ OR ‘foreign workers’ OR ‘domestic worker*’ OR ‘live-in caregiver*’ OR caregiver OR caregivers OR refugee OR refugees* OR asylum) OR AB (Immigrant* OR migrant* OR temporary worker* OR ‘migrant worker*’ OR ‘foreign worker’ OR ‘foreign workers’ OR ‘domestic worker*’ OR ‘live-in caregiver*’ OR caregiver OR caregivers OR refugee OR refugees* OR asylum)
2	(MM “Immigrants+”) OR (MM “Refugees+”)
**3**	**1 OR 2**
**4**	TI (Woman OR women OR female OR females OR adolescent OR adolescents) OR AB (Woman OR women OR female OR females OR adolescent OR adolescents)
7	TI (parturition OR pregnancy OR pregnancies OR pregnant OR gestat* OR Prenatal OR ‘Pre natal’ OR ‘Pre-natal OR ante-natal OR ‘ante natal’ OR antenatal OR ‘peri natal’ OR perinatal OR peri-natal OR ‘post natal’ OR post-natal OR postnatal) OR AB (parturition OR pregnancy OR pregnancies OR pregnant OR gestat* OR Prenatal OR ‘Pre natal’ OR ‘Pre-natal OR ante-natal OR ‘ante natal’ OR antenatal OR ‘peri natal’ OR perinatal OR peri-natal OR ‘post natal’ OR post-natal OR postnatal)
8	TI (illness OR illnesses OR disease OR diseases OR ailed OR suffering OR pain) OR AB (illness OR illnesses OR disease OR diseases OR ailed OR suffering OR pain)
9	TI (‘Childbirth assistance’ OR ‘delivery assistance’ OR ‘assisted delivery’ OR ‘Assisted Parturition’ OR ‘Assisted childbirth) OR AB (‘Childbirth assistance’ OR ‘delivery assistance’ OR ‘assisted delivery’ OR ‘Assisted Parturition’ OR ‘Assisted childbirth)
10	(MH “Prenatal Care”)
11	TI (monitoring OR ‘follow up’ OR exam* OR visit OR ‘education OR treatment OR ‘follow up’ OR follow-up OR Screening OR screen OR test OR tests OR testing OR diagnosis OR care OR consultation OR service OR visit OR diagnosis) OR AB (monitoring OR ‘follow up’ OR exam* OR visit OR ‘education OR treatment OR ‘follow up’ OR follow-up OR Screening OR screen OR test OR tests OR testing OR diagnosis OR care OR consultation OR service OR visit OR diagnosis)
12	TI (Ceasarean OR caesarian OR section OR cesarian OR “cesarean section” OR “caesarean section” OR CS OR C-S OR “C Section” OR C-Section OR “abdominal delivery” OR “abdominal deliveries” OR “cesarean birth” OR “cesarian birth” OR “cesarean deliveries” OR “cesarian deliveries” OR “cesarean delivery” OR “cesarian delivery”) OR AB (Ceasarean OR caesarian OR section OR cesarian OR “cesarean section” OR “caesarean section” OR CS OR C-S OR “C Section” OR C-Section OR “abdominal delivery” OR “abdominal deliveries” OR “cesarean birth” OR “cesarian birth” OR “cesarean deliveries” OR “cesarian deliveries” OR “cesarean delivery” OR “cesarian delivery”)
13	(MH “Childbirth”) OR (MH “Childbirth Education”)
14	(MM “Delivery, Obstetric+”)
**15**	**7 OR 8 OR 9 OR 10 OR 11 OR 12 OR 13 OR 14**
16	TI (Uninsured OR insurance OR ‘without health insurance’ OR ‘Uninsured patient*’) OR AB (Uninsured OR insurance OR ‘without health insurance’ OR ‘Uninsured patient*’)
17	(MM “Insurance, Health+”)
**18**	**12 OR 13**
**19**	**3 AND 4 AND 15 AND 18**
**Web of Science**	
**1**	TOPIC: (Immigrant* OR migrant* OR temporary worker* OR ‘migrant worker*’ OR ‘foreign worker’ OR ‘foreign workers’ OR ‘domestic worker*’ OR ‘live-in caregiver*’ OR caregiver OR caregivers OR refugee OR refugees* OR asylum)
**2**	TOPIC: (Woman OR women OR female OR females OR adolescent OR adolescents)
3	TOPIC: (parturition OR pregnancy OR pregnancies OR pregnant OR gestat* OR Prenatal OR ‘Pre natal’ OR ‘Pre-natal OR ante-natal OR ‘ante natal’ OR antenatal OR ‘peri natal’ OR perinatal OR peri-natal OR ‘post natal’ OR post-natal OR postnatal)
4	TOPIC: (illness OR illnesses OR disease OR diseases OR ailed OR suffering OR pain)
5	TOPIC: (‘Childbirth assistance’ OR ‘delivery assistance’ OR ‘assisted delivery’ OR ‘Assisted Parturition’ OR ‘Assisted childbirth)
6	(MH “Prenatal Care”)
7	TOPIC: (monitoring OR ‘follow up’ OR exam* OR visit OR ‘education OR treatment OR ‘follow up’ OR follow-up OR Screening OR screen OR test OR tests OR testing OR diagnosis OR care OR consultation OR service OR visit OR diagnosis)
8	TOPIC: (Ceasarean OR caesarian OR section OR cesarian OR “cesarean section” OR “caesarean section” OR CS OR C-S OR “C Section” OR C-Section OR “abdominal delivery” OR “abdominal deliveries” OR “cesarean birth” OR “cesarian birth” OR “cesarean deliveries” OR “cesarian deliveries” OR “cesarean delivery” OR “cesarian delivery”)
**9**	**3 OR 4 OR 5 OR 6 OR 7 OR 8 OR 9**
**10**	TOPIC: (Uninsured OR insurance OR ‘without health insurance’ OR ‘Uninsured patient*’)
**11**	**1 AND 2 AND 9 AND 10**
**HINARI**	
**1**	TI (Immigrant* OR migrant* OR temporary worker* OR ‘migrant worker*’ OR ‘foreign worker’ OR ‘foreign workers’ OR ‘domestic worker*’ OR ‘live-in caregiver*’ OR caregiver OR caregivers OR refugee OR refugees* OR asylum) OR AB (Immigrant* OR migrant* OR temporary worker* OR ‘migrant worker*’ OR ‘foreign worker’ OR ‘foreign workers’ OR ‘domestic worker*’ OR ‘live-in caregiver*’ OR caregiver OR caregivers OR refugee OR refugees* OR asylum)
**2**	TI (Woman OR women OR female OR females OR adolescent OR adolescents) OR AB (Woman OR women OR female OR females OR adolescent OR adolescents)
3	TI (parturition OR pregnancy OR pregnancies OR pregnant OR gestat* OR Prenatal OR ‘Pre natal’ OR ‘Pre-natal OR ante-natal OR ‘ante natal’ OR antenatal OR ‘peri natal’ OR perinatal OR peri-natal OR ‘post natal’ OR post-natal OR postnatal) OR AB (parturition OR pregnancy OR pregnancies OR pregnant OR gestat* OR Prenatal OR ‘Pre natal’ OR ‘Pre-natal OR ante-natal OR ‘ante natal’ OR antenatal OR ‘peri natal’ OR perinatal OR peri-natal OR ‘post natal’ OR post-natal OR postnatal)
4	TI (illness OR illnesses OR disease OR diseases OR ailed OR suffering OR pain) OR AB (illness OR illnesses OR disease OR diseases OR ailed OR suffering OR pain)
5	TI (‘Childbirth assistance’ OR ‘delivery assistance’ OR ‘assisted delivery’ OR ‘Assisted Parturition’ OR ‘Assisted childbirth) OR AB (‘Childbirth assistance’ OR ‘delivery assistance’ OR ‘assisted delivery’ OR ‘Assisted Parturition’ OR ‘Assisted childbirth)
6	(MH “Prenatal Care”)
7	TI (monitoring OR ‘follow up’ OR exam* OR visit OR ‘education OR treatment OR ‘follow up’ OR follow-up OR Screening OR screen OR test OR tests OR testing OR diagnosis OR care OR consultation OR service OR visit OR diagnosis) OR AB (monitoring OR ‘follow up’ OR exam* OR visit OR ‘education OR treatment OR ‘follow up’ OR follow-up OR Screening OR screen OR test OR tests OR testing OR diagnosis OR care OR consultation OR service OR visit OR diagnosis)
8	TI (Ceasarean OR caesarian OR section OR cesarian OR “cesarean section” OR “caesarean section” OR CS OR C-S OR “C Section” OR C-Section OR “abdominal delivery” OR “abdominal deliveries” OR “cesarean birth” OR “cesarian birth” OR “cesarean deliveries” OR “cesarian deliveries” OR “cesarean delivery” OR “cesarian delivery”) OR AB (Ceasarean OR caesarian OR section OR cesarian OR “cesarean section” OR “caesarean section” OR CS OR C-S OR “C Section” OR C-Section OR “abdominal delivery” OR “abdominal deliveries” OR “cesarean birth” OR “cesarian birth” OR “cesarean deliveries” OR “cesarian deliveries” OR “cesarean delivery” OR “cesarian delivery”)
9	(MH “Childbirth”) OR (MH “Childbirth Education”)
10	(MM “Delivery, Obstetric+”)
**11**	**3 OR 4 OR 5 OR 6 OR 7 OR 8 OR 9 OR 10**
12	TI (Uninsured OR insurance OR ‘without health insurance’ OR ‘Uninsured patient*’) OR AB (Uninsured OR insurance OR ‘without health insurance’ OR ‘Uninsured patient*’)
13	(MM “Insurance, Health+”)
**14**	**12 OR 13**
**16**	**1 AND 2 AND 11 AND 14**
**Medline (Ovid)**	
1	(Immigrant or immigrants or migrant or migrants or ‘temporary worker’ or ‘temporary workers’ or ‘migrant worker’ or ‘migrant workers’ or ‘foreign worker’ or ‘foreign workers’ or ‘domestic worker’ or ‘domestic workers’ or ‘live-in caregiver*’ or caregiver or caregivers or refugee or refugees or asylum).ab,ti.
2	Refugees.sh.
**3**	**1 OR 2**
**4**	(Woman OR women OR female OR females OR adolescent OR adolescents).ab,ti.
5	(parturition OR pregnancy OR pregnancies OR pregnant OR gestat* OR Prenatal OR ‘Pre natal’ OR Pre-natal OR ante-natal OR ‘ante natal’ OR antenatal OR ‘peri natal’ OR perinatal OR peri-natal OR ‘post natal’ OR post-natal OR postnatal).ab,ti.
6	pregnancy.sh. OR Childbirth.sh
7	(illness OR illnesses OR disease OR diseases OR ailed OR suffering OR pain).ab,ti.
8	(‘Childbirth assistance’ OR ‘delivery assistance’ OR ‘assisted delivery’ OR ‘Assisted Parturition’ OR ‘Assisted childbirth’).ab,ti.
9	(monitoring OR ‘follow up’ OR exam* OR visit OR education OR treatment OR ‘follow up’ OR follow-up OR Screening OR screen OR test OR tests OR testing OR diagnosis OR care OR consultation OR service OR visit OR diagnosis).ab,ti.
10	(Ceasarean OR caesarian OR section OR cesarian OR ‘cesarean section’ OR ‘caesarean section’ OR CS OR C-S OR ‘C Section’ OR C-Section OR ‘abdominal delivery’ OR ‘abdominal deliveries’ OR ‘cesarean birth’ OR ‘cesarian birth’ OR ‘cesarean deliveries’ OR ‘cesarian deliveries’ OR ‘cesarean delivery’ OR ‘cesarian delivery’).ab,ti.
**11**	**5 OR 6 OR 7 OR 8 OR 9 OR 10**
12	(Uninsured OR insurance OR ‘without health insurance’ OR ‘Uninsured patient*’).ab,ti.
13	Insurance.sh.
14	**12 OR 13**
15	**3 AND 4 AND 11 AND 14**
**Embase**	
1	(Immigrant or immigrants or migrant or migrants or ‘temporary worker’ or ‘temporary workers’ or ‘migrant worker’ or ‘migrant workers’ or ‘foreign worker’ or ‘foreign workers’ or ‘domestic worker’ or ‘domestic workers’ or ‘live-in caregiver*’ or caregiver or caregivers or refugee or refugees or asylum).ab,ti.
2	refugee/
**3**	1 OR 2
**4**	(Woman OR women OR female OR females OR adolescent OR adolescents).ab,ti.
5	(parturition OR pregnancy OR pregnancies OR pregnant OR gestat* OR Prenatal OR ‘Pre natal’ OR Pre-natal OR ante-natal OR ‘ante natal’ OR antenatal OR ‘peri natal’ OR perinatal OR peri-natal OR ‘post natal’ OR post-natal OR postnatal).ab,ti.
6	pregnancy/ OR childbirth/
7	(illness OR illnesses OR disease OR diseases OR ailed OR suffering OR pain).ab,ti.
8	(‘Childbirth assistance’ OR ‘delivery assistance’ OR ‘assisted delivery’ OR ‘Assisted Parturition’ OR ‘Assisted childbirth’).ab,ti.
9	(monitoring OR ‘follow up’ OR exam* OR visit OR education OR treatment OR ‘follow up’ OR follow-up OR Screening OR screen OR test OR tests OR testing OR diagnosis OR care OR consultation OR service OR visit OR diagnosis).ab,ti.
10	(Ceasarean OR caesarian OR section OR cesarian OR ‘cesarean section’ OR ‘caesarean section’ OR CS OR C-S OR ‘C Section’ OR C-Section OR ‘abdominal delivery’ OR ‘abdominal deliveries’ OR ‘cesarean birth’ OR ‘cesarian birth’ OR ‘cesarean deliveries’ OR ‘cesarian deliveries’ OR ‘cesarean delivery’ OR ‘cesarian delivery’).ab,ti.
**11**	**5 OR 6 OR 7 OR 8 OR 9 OR 10**
12	(Uninsured or insurance or ‘without health insurance’ or ‘Uninsured patient*’).ab,ti
13	Insurance/
14	**12 OR 13**
15	**3 AND 4 AND 11 AND 14**
**Cochrane Library (Ovid)**	
1	(Immigrant or immigrants or migrant or migrants or ‘temporary worker’ or ‘temporary workers’ or ‘migrant worker’ or ‘migrant workers’ or ‘foreign worker’ or ‘foreign workers’ or ‘domestic worker’ or ‘domestic workers’ or ‘live-in caregiver*’ or caregiver or caregivers or refugee or refugees or asylum):ti,ab,kw
2	MeSH descriptor: [Refugees] explode all trees
**3**	**1 OR 2**
**4**	(Woman OR women OR female OR females OR adolescent OR adolescents):ti,ab,kw
5	(parturition OR pregnancy OR pregnancies OR pregnant OR gestat* OR Prenatal OR ‘Pre natal’ OR Pre-natal OR ante-natal OR ‘ante natal’ OR antenatal OR ‘peri natal’ OR perinatal OR peri-natal OR ‘post natal’ OR post-natal OR postnatal):ti,ab,kw
6	MeSH descriptor: [pregnancy] explode all trees
7	(illness OR illnesses OR disease OR diseases OR ailed OR suffering OR pain):ti,ab,kw
8	(‘Childbirth assistance’ OR ‘delivery assistance’ OR ‘assisted delivery’ OR ‘Assisted Parturition’ OR ‘Assisted childbirth’):ti,ab,kw
9	(monitoring OR ‘follow up’ OR exam* OR visit OR education OR treatment OR ‘follow up’ OR follow-up OR Screening OR screen OR test OR tests OR testing OR diagnosis OR care OR consultation OR service OR visit OR diagnosis):ti,ab,kw
10	(Ceasarean OR caesarian OR section OR cesarian OR ‘cesarean section’ OR ‘caesarean section’ OR CS OR C-S OR ‘C Section’ OR C-Section OR ‘abdominal delivery’ OR ‘abdominal deliveries’ OR ‘cesarean birth’ OR ‘cesarian birth’ OR ‘cesarean deliveries’ OR ‘cesarian deliveries’ OR ‘cesarean delivery’ OR ‘cesarian delivery’):ti,ab,kw
**11**	**5 OR 6 OR 7 OR 8 OR 9 OR 10**
12	(Uninsured OR insurance OR ‘without health insurance’ OR ‘Uninsured patient*’):ti,ab,kw
13	MeSH descriptor: [Insurance] explode all trees
**14**	**12 OR 13**
15	**3 AND 4 AND 11 AND 14**
**Scopus**	
1	(uninsured OR insurance) AND (migrant OR immigrant OR refugee* OR asylum) AND (women OR woman OR female OR adolescent*) AND (parturition OR pregnancy OR pregnancies OR pregnant OR gestat*) AND (prenatal OR ‘pre natal’ OR pre-natal OR ‘ante natal’ OR antenatal OR ‘peri natal’ OR perinatal OR ‘post natal’ OR post-natal OR postnatal)
**ScienceDirect**	
1	(uninsured OR insurance) AND (migrant OR immigrant) AND (women OR adolescent) AND (antenatal OR postnatal OR prenatal)

### Policy dynamics and system responsiveness

Findings from the included studies indicate that policy environments are dynamic and responsive to migration-related pressures. In North America, federal-level restrictions have, in some cases, shifted responsibility to state or provincial governments to adapt eligibility criteria. For example, policy changes following Bill Clinton’s 1996 declaration [61] have contributed to variability in access across jurisdictions. This decentralised flexibility can represent a strength, as some regions have expanded access to perinatal care for PMWMIs [57], potentially reducing costly emergency interventions [57].

At the same time, variability across jurisdictions may also constitute a weakness, leading to unequal access depending on geographic location. Broader contextual evidence indicates that migration flows have increased in recent years in countries such as Canada and the United States [62, 63], placing additional pressure on healthcare systems and influencing policy evolution. While this trend is not derived directly from the included studies, it provides important context for understanding the policy dynamics observed in the findings.

Some studies also describe collaborative arrangements between host countries and international organisations [64], supported by global frameworks such as the New York Declaration [55], to improve access to care for migrant populations. These examples illustrate both adaptive policy responses and ongoing inconsistencies in implementation.

**Economic relevance of policies**

From a cost perspective, included studies suggest that restrictive policies may create the appearance of short-term savings while generating higher long-term costs. Evidence indicates that lack of access to timely prenatal care leads to more complex and expensive interventions later, including increased postnatal care costs [52]. In addition, evidence suggests that even in high-income countries with adequate resources, the provision of services for PMWMIs remains insufficient [65], highlighting a significant gap in healthcare systems. NGOs play a critical role in addressing these gaps [66], representing an important strength of current systems; however, their limited capacity also highlights a structural weakness, as they cannot fully substitute for comprehensive public coverage.

Broader interpretations of these findings may be informed by theoretical perspectives such as the Womb Theory and Early Childhood Theory [67, 68], which emphasise the long-term benefits of early-life health investments. While these theories were not explicitly examined in the included studies, they provide a useful framework for interpreting the economic implications of restricted access to care.

### Policy lag and access gaps

The included studies indicate that policy adaptation often lags behind changes in migration patterns, creating gaps in access to care. During these periods, NGOs frequently act as interim providers, addressing urgent needs among uninsured migrant populations. Broader contextual examples, such as recent migration trends -including increased arrivals in the United States [69] and events such as the Lampedusa tragedy [70–72] further illustrate how sudden population movements can place pressure on healthcare systems, although these examples extend beyond the included studies.

Evidence from the included literature also suggests that institutional practices, such as hospital billing policies for uninsured patients [73], may restrict access to necessary services. These findings highlight a key weakness in current systems: the lack of timely policy responsiveness. Given the vulnerability associated with perinatal care, some studies recommend the development of proactive and targeted systems to support high-risk groups, including asylum seekers and temporary migrants [65].

### Ethical and public health implications

Findings from the included studies raise important ethical and public health concerns related to restricted access to perinatal care. For example, Emergency Medicaid in the United States provides coverage only for life-threatening conditions or childbirth, reaching a limited proportion of undocumented migrants [74]. This represents a significant limitation in continuity and equity of care.

From a public health perspective, the exclusion of PMWMIs from routine screening and preventive services may increase population-level risks, particularly for communicable diseases such as HIV/AIDS [27]. National initiatives, such as the Canadian Perinatal HIV Surveillance Program [75], highlight the importance of inclusive screening policies in mitigating these risks.

Some findings also indicate that alternative models of care delivery, such as home visits [57], may improve access among undocumented populations who fear engagement with formal institutions. Community-based approaches and partnerships with local organisations may therefore represent effective strategies for strengthening access to care. The findings of this review underscore the need for inclusive policies that guarantee access to essential perinatal services for PMWMI. Locally, expanding publicly funded prenatal care, reducing administrative barriers, and integrating community‑based organizations into formal care pathways could reduce preventable maternal and neonatal complications. Globally, these results align with international commitments promoting universal health coverage and migrant health equity. Strengthening rights‑based approaches and harmonizing national policies with global frameworks would support more consistent access to perinatal care and reduce disparities across health systems [76].

### Implementation challenges

The included studies identify several barriers to the effective implementation of policies aimed at improving access to care for PMWMIs. These include the complexity of legal frameworks, variability in interpretation by providers, and administrative barriers related to reimbursement systems. Such challenges may discourage providers from offering services, even where policies formally allow access [13].

Importantly, evidence suggests that possession of insurance coverage does not always guarantee access to care. For example, Lusambili et al. [13] report that refugee women in Kenya were denied services despite holding valid insurance cards. These findings underscore the gap between policy design and practical implementation.

### Strengths and limitations

This review included a limited number of studies (n = 13), primarily from four countries (Canada, the United States, Iran, and Thailand), which may limit generalisability. In addition, evolving policy contexts and eligibility criteria introduce variability across studies. The restriction to English and French publications may also have resulted in the exclusion of relevant studies. This limitation reflects resource and translation constraints within the review team and may have narrowed the evidence base by excluding studies published in other languages. Furthermore, the predominance of studies from high‑income countries (85%) requires caution in interpreting the results for low‑ and middle‑income settings, where migration patterns and health system contexts may differ.

However, the use of a scoping review methodology enabled the inclusion of diverse study designs and grey literature, providing a comprehensive overview of available evidence. The systematic approach to data extraction and synthesis strengthens the robustness of the findings. Additionally, the cost‑related findings reported in the included studies should be interpreted with caution, as the economic estimates are context‑specific and may not be generalisable across different health systems or policy environments. Variability in underlying assumptions and cost structures further limits the comparability of these cost‑benefit claims.

### Novelty statement

This scoping review represents the first focused synthesis of interventions addressing access to perinatal care for PMWMIs. The findings highlight persistent gaps in access to care despite ongoing policy developments in high-income countries. While some progress has been observed, such as the reinstatement of universal health coverage in Spain in 2018 following earlier restrictions [77], access remains inconsistent, particularly in contexts where policies fluctuate in response to political priorities, as observed in the United States.

Overall, this review underscores the need for more consistent, inclusive, and evidence-informed policies to improve equitable access to perinatal care for migrant populations.

## Conclusion

The gaps created by policy changes, such as the reduction of services, foster an illusion of economic efficiency. While they may appear to yield short-term savings, they often result in greater expenses in the medium to long term. This burden is shifted to the states and provinces, which are compelled to compensate for the shortfall. The outcomes indicate that these changes appear to be politically motivated rather than economically beneficial.

From a practical perspective, our findings highlight the importance of prioritizing interventions and programs that ensure access to perinatal health services for PMWMI. Investing in such programs is likely to reduce overall costs for taxpayers over time while improving health outcomes for this vulnerable population.

From a research perspective, we recommend conducting contextual impact analyses of interventions and policies that promote or restrict access to perinatal care for PMWMIs. Such analyses could inform more effective, equitable, and economically sound policy decisions

## Supporting information

S1 Checklist Preferred Reporting Items for Systematic reviews and Meta-Analyses extension for Scoping Reviews (PRISMA-ScR) Checklist. (DOCX)

S2 Appendix Charted data extracted from the included studies. (XLSX)
