# Supplementary material for: Interventions facilitating access to perinatal care for migrant women without medical insurance: a scoping review
Source: PLoS One. 2026 Sep 10;21(9):e0357487. doi: 10.1371/journal.pone.0357487 (PMC13561353; doi:10.1371/journal.pone.0357487)
Supplement: Supplementary file 2 [file pone.0357487.s004.docx]

Response to **the Editor**

| 1.Please ensure that your manuscript meets PLOS ONE's style requirements, including those for file naming. The PLOS ONE style templates can be found at  <https://journals.plos.org/plosone/s/file?id=wjVg/PLOSOne_formatting_sample_main_body.pdf> and  [https://journals.plos.org/plosone/s/file?id=ba62/PLOSOne_formatting_sample_title_authors_affiliations.pdf](https://can01.safelinks.protection.outlook.com/?url=https%3A%2F%2Ftrack.editorialmanager.com%2FCL0%2Fhttps%3A%252F%252Fjournals.plos.org%252Fplosone%252Fs%252Ffile%253Fid%3Dba62%252FPLOSOne_formatting_sample_title_authors_affiliations.pdf%2F1%2F010f019c2186e9e3-e31ffff1-c153-411f-a526-201d96b0662f-000000%2Ft-ad8jVqBemhVqNNCMvgS8k6zaJo-07HsCxFfu4JHjs%3D248&data=05%7C02%7Cibeogo%40uottawa.ca%7C1ff36f475f8d4bd5eef108de89e28d19%7Cd41fdab17e154cfdb5fa7200e54deb6b%7C1%7C0%7C639099803747061605%7CUnknown%7CTWFpbGZsb3d8eyJFbXB0eU1hcGkiOnRydWUsIlYiOiIwLjAuMDAwMCIsIlAiOiJXaW4zMiIsIkFOIjoiTWFpbCIsIldUIjoyfQ%3D%3D%7C0%7C%7C%7C&sdata=Lq9Onjud%2BCXW0Co9aJc9RMrPywb1ju%2FpX1EvaIgQOx4%3D&reserved=0) | Thank you for this comment. We carefully reviewed and revised the manuscript to ensure compliance with PLOS ONE style and formatting requirements. |
| --- | --- |
| 2. Please note that funding information should not appear in any section or other areas of your manuscript. We will only publish funding information present in the Funding Statement section of the online submission form. Please remove any funding-related text from the manuscript. | Thank you for this comment. We have carefully reviewed the manuscript and removed all funding-related information from the main text |
| 3. Thank you for stating the following financial disclosure:  “SHERPA University Institute and the Social Sciences and Humanities Research Council (SSHRC) have funded this project”  Please state what role the funders took in the study. If the funders had no role, please state: "The funders had no role in study design, data collection and analysis, decision to publish, or preparation of the manuscript."  If this statement is not correct you must amend it as needed.  Please include this amended Role of Funder statement in your cover letter; we will change the online submission form on your behalf. | **The funding statement was removed**  For the role of the founder, the following excerpt was added  **Role of the funding agency**  The funders had no role in study design, data collection and analysis, decision to publish, or preparation of the manuscript. |
| 4. Please provide a complete Data Availability Statement in the submission form, ensuring you include all necessary access information or a reason for why you are unable to make your data freely accessible. If your research concerns only data provided within your submission, please write "All data are in the manuscript and/or supporting information files" as your Data Availability Statement. | Because data is provided within our submission, we now stated it as follow:  All data are in the manuscript and/or supporting information files" |
| 5. Please include a copy of Table 3 which you refer to in your text on page 8. | Thank you for your comment. Table 3 has now been included in the manuscript. Unlike the table presented in the published protocol, which included the search strategy for a single database, this expanded table presents the complete search strategies for all databases consulted in the review. |
| 6. If the reviewer comments include a recommendation to cite specific previously published works, please review and evaluate these publications to determine whether they are relevant and should be cited. There is no requirement to cite these works unless the editor has indicated otherwise | Thank you for this comment. Although no specific additional references were requested by the reviewers, we carefully reviewed the literature and incorporated additional relevant references where appropriate to strengthen the manuscript. |
| Intro - Please provide references for assertions made throughout. For example: “Four out of five uninsured pregnant women received inadequate prenatal care. More than half of these women received insufficient prenatal care, and 6.5% received no prenatal care at all.” – What are the references for these? | Sorry for that for the lack of the reference.  The statement is supported by Perinatal outcomes of uninsured immigrant, refugee and migrant mothers and newborns living in Toronto, Canada, which reports that four in five uninsured women received inadequate prenatal care, over half received clearly inadequate care, and 6.5% received none.  Wilson-Mitchell K, Rummens JA. Perinatal Outcomes of Uninsured Immigrant, Refugee and Migrant Mothers and Newborns Living in Toronto, Canada. International Journal of Environmental Research and Public Health 2013;10:2198-213. |
| - “Insurance status was also linked to the type of health care provider, reasons for caesarean section, neonatal resuscitation rates, and the length of maternal hospital stay.” Was linked in what study? In what country? More transparent information required here. | Thank you for this comment. We have used this revision as an opportunity to refine the excerpt, which now reads as follows:  “Similar patterns have been documented internationally, where immigrant women frequently encounter inadequate prenatal care (6-9), contributing to higher stillbirth rates (10). Those lacking medical insurance often initiate care late in pregnancy and facing more severe delivery complications (11), including more emergency caesarean sections, which can result in lasting trauma for both mother and child (11)”. |
| - The argument in the intro is also confusing as contradictory in parts. For example, you assert: “…many migrant women in Canada and the United States often receive care that falls below recommended standards due to a lack of public health insurance coverage (6)”, but then immediately after (lines 11-15) you note that in Canada most immigrants and resettled refugees do have universal coverage comparable to citizens. Without qualification, those two claims directly clash. | To avoid any misunderstanding, we removed the initial sentence to focus more directly on the substance of the argument concerning refugees and temporary migrants  Please see, line01 to 28 |
| - The population of interest is not clearly defined and appears inconsistent across the title and background. While the title and text refer broadly to “migrant women,” the background indicates that immigrants and resettled refugees in Canada have universal health coverage, whereas lack of insurance applies specifically to undocumented migrants and certain temporary visa holders. These groups are not interchangeable.  - The authors should clearly specify which migrant subgroups are the focus of the review, ensure alignment between the title, aims, and background, and avoid using “migrant women” as a blanket term if the focus is on uninsured subpopulations. | **Thank you for this very pertinent comment, which has significantly contributed to improving the quality of our revised manuscript. In light of your observation, we have implemented the following changes:**   - **We now use the term** migrant **consistently throughout the manuscript, replacing** immigrant **where appropriate to ensure conceptual clarity.** - **Although the populations referenced differ, they all experience considerable vulnerability, particularly in terms of access to the health‑care system. In revising the background section, we sought to better highlight these nuances and clarify how each group benefits—or fails to benefit—from available health‑care coverage.**   **Overall, our primary focus remains on migrant women without health insurance. Refugees represent a specific subgroup: despite the protections granted under the Geneva Convention and the coverage to which they are theoretically entitled, their access to health services and the quality of the care they receive often remain suboptimal.** |
| - There is also inconsistency in the use of “perinatal care.” While the review claims to examine perinatal interventions, the background and epidemiological data presented relate almost exclusively to antenatal care. If postnatal and intrapartum care are within scope, this should be reflected in the background and rationale. Otherwise, terminology should be tightened and used consistently from the outset. | Thank you for your comment. We adopted a broad approach to include prenatal care, perinatal care (for example, emergency cesarean sections), and morbid events occurring during the postnatal period, such as postpartum depression.  Indeed, the concept of perinatal care encompasses prenatal, intrapartum, and postnatal care, as defined by the World Health Organization.In this review, prenatal care is considered an integral part of perinatal interventions. |
| Materials and Methods - The sentence “This study utilized the Arksey and O’Malley” is incomplete as written and should be revised for clarity. | It is now frame as follow:  This study utilized the Arksey and O’Malley framework (26). |
| - The research questions and overarching aim should be presented at the end of the Introduction, not in the Methods section. | It’s done accordingly |
| - The research questions require clarification. Research question 2 combines multiple distinct elements (impacts, strengths, weaknesses, and costs), which reduces conceptual clarity. The authors should consider streamlining or separating this question. In addition, “policies” should be clearly positioned within the scope of the review, for example as a type of intervention, and the terminology used consistently. | We thank the reviewer for this constructive comment. In response, we have revised the research questions to improve conceptual clarity and analytical coherence. Specifically, the original second research question, which combined intervention impacts, strengths, weaknesses, and costs, has been streamlined and separated conceptually. The revised research questions are now clearly articulated. |
| - For the inclusion criteria, please clarify whether a cut-off period was applied for the postpartum period.  - The inclusion criteria are unclear and partially redundant. The perinatal period is already defined by pregnancy, labour, and postpartum; the subsequent sentence referring again to “articles addressing the perinatal period” adds no additional clarification and creates ambiguity. Please streamline and clearly define the perinatal timeframe used for study inclusion. | We are sorry for this lack of clarity that we noticed does not appear in the submitted version. We now streamline the text and put more light on the inclusion criteria.  As presented in the protocol (34), we used the World Health Organisation operational definitions. That is, details on the cut-off are now mentioned with more clarity including all types of services.  Please see on p9, line 137 to 142 |
| - Lines 75-85 under “Population”: This material is conceptual and definitional and would be more appropriately located in the Introduction. Clarification of who is encompassed by the term “migrant” (and which subgroups are within scope) is necessary for framing the review, rather than being embedded in the Methods section. The population of interest should be clearly defined upfront and aligned with the title and aims. | Thank you for this insightful comment. We have revised the manuscript to relocate the conceptual and definitional discussion of “migrant” from the Methods section to the Introduction, providing a clearer context for the review. Additionally, we have explicitly defined the population of interest. These revisions ensure that the study population is clearly identified from the outset and that the manuscript’s terminology aligns with the title and objectives. Overall, these changes enhance the clarity, coherence, and framing of the review. |
| - The content presented under “Interventions” primarily describes the population and perinatal timeframe rather than defining the intervention concept. This section should clearly specify what was considered an intervention (including policy-level interventions), what was excluded, and the rationale for these decisions. Population and timeframe definitions should be addressed in the intro and briefly in “Population” and kept distinct from intervention eligibility. | Thank you for this insightful comment. We have revised the “Interventions” section to clearly define the intervention components, specifying the maternal and newborn health services included—such as pregnancy testing, prenatal care and follow-up, screening for diseases, immediate obstetric and neonatal care, postpartum and newborn care, prenatal education, vaccination, and childbirth assistance—as well as policy-level interventions designed to support and sustain these services. We have also added a statement clarifying which services were outside the scope of the intervention and the rationale for these exclusions. |
| - In PICO, the comparator would be another intervention, usual care, or explicitly “no comparator”. The existing content presented under “Comparator” is not appropriate. This section describes study design, language, and publication eligibility rather than a comparator. | Thank you for this comment. We acknowledge that the original text in the “Comparator” section did not align with the PICO framework. We have revised the section to explicitly state that no comparator was anticipated in this review. |
| - The Outcomes section is incorrectly framed. Rather than describing what the review reported, this section should specify the outcome domains considered for study inclusion. So, studies were included if.. | Thank you. We revised the “Outcomes” section to include studies reporting intended results, such as maternal and newborn health services or supporting policy interventions. |
| - The search strategy requires clarification. The section refers to “eleven electronic databases” but then lists journals, reference lists, and organisational websites, which are not databases. In addition, the inclusion of NGO and organisational websites appears inconsistent with the stated eligibility criteria limiting inclusion to empirical scientific studies, unless reports or grey literature were also included. The authors should clearly distinguish between databases and other sources, clarify what types of records were eligible, and ensure consistency with the stated inclusion criteria. | Thank you. We have clarified the search strategy by distinguishing the eleven electronic bibliographic databases (CINAHL, Web of Science, Medline-Ovid, Embase, Cochrane Library, Scopus, ScienceDirect, Hinari, Lilacs, Cairn, BDSP) from additional sources, including reference lists, key journals (Revue Migrations Forcées, Migrations Société), and relevant NGO websites (Médecin du Monde, MSF, UNHCR). |
| - Importantly: The authors note that the search was last updated in December 2024. Given that more than 12 months have elapsed, the search should be updated to ensure the review reflects the current evidence base. | We update on April 16, 2026, and end up with one more study (Kim and al. 2025) |
| - The PICO elements should be presented within the Study selection section, rather than preceding it. | Thank you. We have revised the manuscript to present the PICO elements within the “Study Selection” section, rather than preceding it, ensuring alignment with journal guidelines and improving the logical flow of the review. |
| - The data analysis section lacks methodological clarity. The authors describe general activities (e.g. categorising studies, summarising findings) rather than specifying the analytic approach used. Please clarify the analytical method applied (e.g. descriptive mapping, thematic synthesis, framework-based analysis), including how data were compared and synthesised. | Thank you. We have revised the Data Analysis section to clarify the methodological approach. Data were analysed using a narrative synthesis framework: studies were categorized by objectives, and findings were compared across interventions and settings. Key outcome domains—including impact, strengths, weaknesses, and costs—were extracted and synthesized thematically to identify patterns and implications for research, practice, and policy, providing a structured and transparent approach to summarizing evidence. |
| Results - The Results section should begin with a clear descriptive numerical summary of the included studies (e.g. total number of studies, countries, study designs, populations), consistent with scoping review reporting standards. Currently, the section opens with an interpretive statement rather than a structured overview. | We thank the reviewer for this valuable observation. In response, we have revised the Results section to begin with a structured descriptive numerical summary of the included studies, consistent with scoping review reporting standards. The revised section now presents an overview of the total number of included studies, study locations, study designs, and populations prior to any interpretive or thematic analysis. This modification improves clarity, transparency, and alignment with PRISMA‑ScR guidance. |
| - The rationale for the organisation of results under the current headings (e.g. “Interventions in favor of access…”, “Actors involved…”) is unclear. The authors should explicitly state the basis for this structure (e.g. analytic framework, thematic synthesis, a priori categories) and ensure it aligns with the described data analysis approach. | We thank the reviewer for this important observation. In response, we have clarified the analytical basis underpinning the organisation of the Results section. Specifically, the results are structured according to a thematic analysis, in which themes emerged inductively from the data, but the organisation of the Results section was guided **by key outcome domains** relevant to the review objectives, including intervention impacts, strengths, weaknesses, and costs. The current headings represent key analytical themes that emerged through multiple rounds of discussion and consensus within the research team, rather than a priori categories.  We have revised both the Methods section accordingly. |
| - Overall, the Results section lacks coherence and a clear analytic logic. The basis for organising results under the current headings is not specified and does not clearly align with a stated analytical framework or method. It is unclear whether findings are being grouped by intervention type, policy level, actor, outcome, or study focus, and the structure appears to shift across sections without justification. | We thank the reviewer for this detailed and constructive comment. In response, we have strengthened the clarity and coherence of the analytical logic underpinning the Results section and explicitly aligned its structure with the revised data‑analysis approach.  As clarified in the Methods section, the analysis was conducted using a narrative synthesis informed by thematic analysis, whereby findings were thematically synthesized across study objectives, intervention types, and settings. While themes emerged inductively through this process, the presentation of results was deliberately organised around key outcome domains aligned with the review objectives, namely intervention impacts, strengths, weaknesses, and costs. This approach was adopted to ensure a consistent and transparent organisational logic across sections. |
| - the section titled “Strengths and weaknesses of these interventions” is unclear. It is not specified whether this refers to the strengths and weaknesses of the interventions themselves, the policies, or the quality of the included studies. As presented, this section conflates empirical findings with interpretation and lacks methodological clarity. | We thank the reviewer for this important observation. In response, we have clarified the scope and analytical intent of the section previously titled “Strengths and weaknesses of these interventions.” The section has been renamed “Strengths and weaknesses of perinatal care policies and their implementation” to more accurately reflect its focus.  We clarify that this section synthesizes empirical findings related to policy‑level and programmatic interventions and their implementation, rather than assessing the methodological quality of the included studies. The strengths and weaknesses discussed refer specifically to how perinatal care policies and reforms have facilitated or hindered access to care for PMWMIs, as reported in the included studies, including aspects of policy design, implementation processes, and their documented consequences.  To improve methodological clarity, we have added a brief framing sentence at the beginning of the section to delineate its analytical scope and ensure consistency with the stated thematic synthesis approach. These revisions enhance transparency and strengthen coherence across the Results section. |
| - The authors should: – Clearly state the analytic approach used to structure and synthesise results; | We thank the reviewer for this important observation. In response, we have clarified the analytical basis underpinning the organisation of the Results section. Specifically, the results are structured according to a thematic analysis, in which themes emerged inductively from the data, but the organisation of the Results section was guided by **key outcome domains** relevant to the review objectives, including intervention impacts, strengths, weaknesses, and costs. The current headings represent key analytical themes that emerged through multiple rounds of discussion and consensus within the research team, rather than a priori categories.  We have revised both the Methods section accordingly. |
| – Ensure that section headings are conceptually precise and consistently applied; | We thank the reviewer for this detailed and constructive comment. In response, we have strengthened the clarity and coherence of the analytical logic underpinning the Results section and explicitly aligned its structure with the revised data‑analysis approach.  As clarified in the Methods section, the analysis was conducted using a narrative synthesis informed by thematic analysis, whereby findings were thematically synthesized across study objectives, intervention types, and settings. While themes emerged inductively through this process, the presentation of results was deliberately organised around key outcome domains aligned with the review objectives, namely intervention impacts, strengths, weaknesses, and costs. This approach was adopted to ensure a consistent and transparent organisational logic across sections. |
| – Distinguish clearly between results, interpretation, and discussion-level critique. | We thank the reviewer for this important comment. While analytical synthesis is inherent to thematic scoping reviews, we have further refined the Results section to strengthen the distinction between empirical findings, analytical synthesis, and discussion‑level interpretation.  Specifically, we revised Sections 3 and 4 to ensure that the Results section reports findings strictly as documented in the included studies, with all statements explicitly grounded in reported evidence and attributed accordingly. Evaluative language and broader interpretive commentary have been removed from the Results section and reserved for the Discussion. These revisions reinforce a clear separation between results and discussion‑level critique, consistent with scoping review reporting conventions. |
| **Discussion** - The Discussion section requires tighter alignment with the aims, research questions, and findings of the review. Several sections (e.g. ethical implications, economic theory, policy agility) introduce themes that were not clearly framed in the Introduction nor explicitly examined in the Results. As written, it is often unclear whether these points are derived from the included studies or represent broader commentary.  - The authors should refocus the Discussion on interpreting the review’s findings, clearly link all discussion points to the included evidence, and avoid introducing new conceptual themes that fall outside the stated scope of the review. | We thank the reviewer for this important and constructive comment. In response, we have revised the Discussion section to ensure tighter alignment with the stated aims, research questions, and empirical findings of the review.  Specifically, we clarified which discussion points are directly derived from the included studies and which represent interpretive synthesis grounded in the review findings. Sections that introduced broader considerations (e.g., ethical implications, economic considerations, and policy adaptability) have been revised to explicitly link these discussions to results reported in the included studies. Where such connections could not be clearly established, content has been either reframed, relocated, or removed.  We have also streamlined the Discussion to focus on themes examined in the Results section. These revisions improve conceptual coherence and transparency and align the Discussion more closely with the evidence synthesized in the Results. |

Response to **Reviewer 1**

| For the strengths, the use of multiple databases, grey literature, NGO websites, and hand-searching increases the review’s breadth. The involvement of an experienced librarian adds rigor to the search strategy. Furthermore, the selection process is clear with a well-structured data extraction strategy. - Lines two to five should have a reference | Thank you for your observation. We have now added the appropriate reference to support the statements. The revised manuscript includes this citation to ensure clarity and proper attribution. |
| --- | --- |
| - From line 11 to 31 is such a long paragraph, can be made two paragraphs | Thank you for the helpful suggestion. We have revised the manuscript accordingly by dividing the long passage into two clearer paragraphs. This change improves readability and strengthens the overall structure of the section. |
| - The review is heavily dominated by studies from the USA and Canada (85%), limiting the generalizability to global migrant contexts especially low and middle-income countries where migration dynamics differ. Hence, they authors could indicate interpretation with cognisance of this | Thank you for highlighting this important point. We have now clarified that this concentration of evidence requires caution when interpreting the findings for low‑ and middle‑income countries, where migration patterns and health system contexts may differ. This revision has been incorporated into the manuscript to ensure the interpretation reflects this contextual limitation. |
| - The acronym PMWMIs is used for “pregnant migrant women without medical insurance,” but later appears to refer to postpartum women with mental illness on line 254—this should be corrected for consistency. | Thank you for pointing this out. It is a typo. The acronym PMWMIs is intended to refer exclusively to *pregnant migrant women without medical insurance*. The inconsistent use identified has been corrected to ensure accurate and consistent terminology throughout the manuscript. |

Response to **Reviewer 2**

Thank you. This was an interesting read. The work aligned with global public health priorities, and was quite timely. This reviewer only has some minor comments.

| 1. Limiting to English and French would, undoubtedly, limit generalizability. There would need to be a stronger justification, and preferably a sensitivity analysis. | Thank you for this observation. The manuscript now provides a clearer justification for limiting the review to English and French. This constraint reflects available translation resources, and its potential to narrow the evidence base is explicitly acknowledged in the revised text. |
| --- | --- |
| 2. With a rather limited number of existing evidence, the authors could also consider expanind to the grey literature. | Thank you for this helpful suggestion. The review already incorporated grey literature as part of the search strategy. This included targeted searches of organisational reports and non‑peer‑reviewed sources, namely in websites of non-governmental organizations (NGOs) such as Médecins du Monde, Médecins Sans Frontières (MSF), and the United Nations High Commissioner for Refugees (UNHCR). This has now been clarified in the manuscript (***Data sources and research strategy***) to emphasise that grey literature was actively considered and integrated into the evidence base. |
| 3. What was the screening process? Was there a reliability count? How exactly was the "discussion" done - the conflicts may best be quantified. | Thank you for this comment. The study selection process is detailed in the section “Studies Screening.” Screening followed a structured, multi‑step approach. After duplicate removal, 10% of records were jointly reviewed using the IED‑uOttawa algorithm to harmonize decisions. Titles and abstracts were then independently screened by three reviewers.  Across this stage, 2.32% of screening decisions resulted in a conflict, all of which were resolved through discussion after re‑examining the record. Based on this conflict rate, the implied agreement between reviewers corresponds to 97.68%, reflecting strong consistency in the application of eligibility criteria. When consensus could not be reached, a fourth reviewer adjudicated.  Full‑text screening was conducted independently by two reviewers, followed by a group meeting to document exclusion reasons and finalize the flow diagram.  The section was reshaped accordingly. |
| 4. Was there any quality appraisal for the included studies? | Thank you for this question. As this is a scoping review, no formal quality appraisal of included studies was conducted, in accordance with the Arksey and O’Malley framework and current scoping review methodology guidelines. The objective was to map the breadth of available evidence rather than assess study quality or risk of bias. |
| 5. Was it possible to deepend the synthesis and analysis? Like a thematic analysis? | Thank you for this helpful comment. In response, we have clarified the analytical approach in the revised manuscript. Data analysis was conducted using a narrative synthesis informed by thematic analysis. Following data extraction, study findings were compared across study objectives, intervention types, and settings, and recurring patterns were identified through iterative review and discussion within the research team.  Although themes emerged inductively, the Results section is organised around outcome domains aligned with the review objectives—intervention impacts, strengths, weaknesses, and costs—to ensure a coherent and transparent presentation. This combined approach allowed us to deepen the synthesis while remaining consistent with the methodological aims of a scoping review and to identify strategies that support access to perinatal care for PMWMIs, as well as implications for research, practice, and policy. |
| 6. The consultation process was mentioned, but more details should be reported. | Thank you for this comment. The consultation phase described in the Arksey and O’Malley framework is optional, and we did not undertake this step in the present review. We have now clarified this explicitly in the Methods section. |
| 7. Uncritically presented cost-benefit claims may not be the best way forward. Please consider applying more critial discussions, like inflation adjustments or generalizability. | Thank you for this insightful comment. We have addressed it by adding a more critical reflection on the cost‑benefit findings in the limitations section. Specifically, we now note that the economic estimates reported in the included studies are context‑specific, rely on varying assumptions, and may not be generalisable across different health systems or policy environments. This addition ensures that the cost‑related claims are interpreted with appropriate caution. |
| 8. There seemed to be some minor formatting and typo issues. | Thank you for noting the formatting and typographical issues. We have carefully reviewed the manuscript and corrected minor errors, including inconsistencies in formatting, punctuation, and repeated text. These revisions improve the clarity and readability of the manuscript. |
| 9. "First of its kind" was rather a strong claim. Please consider citing prior reviews on migrant maternal health and differentiate. | Thank you for this helpful comment. We agree that the original phrasing overstated the novelty of our review. We have removed the “first of its kind” claim and now cite prior reviews on migrant maternal health, including Stevenson et al. (2024), Gieles et al. (2019), and Heslehurst et al. (2018). We also clarify how our review differs from this existing work: whereas previous reviews examined migrant maternal health more broadly or focused on clinical outcomes among asylum seekers and undocumented migrants, none specifically synthesized evidence on interventions designed to facilitate access to perinatal care for migrant women without medical insurance or with precarious migration status. Our review addresses this gap by focusing on this particularly underserved subgroup. |
| 10. Please discuss more on implications on local and global policies. | Thank you for this valuable suggestion. We have expanded the Discussion to more fully address the implications of our findings for both local and global policy. Specifically, we added a dedicated paragraph outlining how inclusive health‑coverage policies, reduced administrative barriers, and strengthened community partnerships can improve access at the local level. We also discuss how our results align with global commitments to universal health coverage and migrant health equity, highlighting the relevance of rights‑based approaches and international frameworks such as the WHO Global Action Plan on Refugee and Migrant Health. These additions clarify the broader policy significance of our review. |

Response to **Reviewer 3**

| **Reviewer #3**: This manuscript presents a well-conducted and timely scoping review addressing a critically important and underexplored area of maternal and public health: access to perinatal care for migrant women without medical insurance. The topic is highly relevant in the context of increasing global migration and persistent health inequities affecting undocumented migrants and asylum seekers. The authors have applied the Arksey and O’Malley framework appropriately and transparently, ensuring methodological rigor throughout the scoping review process. The inclusion of both peer-reviewed literature and grey literature strengthens the comprehensiveness of the findings and enhances the policy relevance of the study. The clear description of search strategies, inclusion criteria, and study selection adds to the credibility and reproducibility of the review. The synthesis of evidence across multiple national contexts (Canada, the United States, Iran, and Thailand) is a notable strength, as it allows for meaningful cross-country comparisons and highlights how different health system structures influence access to perinatal care. The discussion effectively links policy changes to their short-, medium-, and long-term economic and health consequences, offering valuable insights into how restrictive policies may inadvertently increase healthcare costs and worsen health outcomes. The manuscript is clearly written, logically structured, and accessible to a broad readership, including researchers, clinicians, and policymakers. The conclusions are well supported by the presented evidence and successfully underscore the public health and ethical implications of excluding migrant women from perinatal care. Importantly, the authors’ emphasis on political drivers of policy decisions adds depth to the analysis and contributes to ongoing discussions around health equity and social justice. | We thank Reviewer for their thoughtful and encouraging comments. We appreciate the recognition of the relevance of our topic and the methodological rigor of our scoping review. We are pleased that the reviewer found the search strategy, study selection process, and cross‑country synthesis to be clear and valuable. We also welcome the reviewer’s acknowledgement of the contribution our analysis makes to ongoing discussions on health equity, policy, and social justice for migrant women without medical insurance. |
| --- | --- |

Response to **Reviewer 4**

| Abstract: a. Materials and Methods: Kindly include the rationale for using the Arksey and O’Malley (2005) framework in the Abstract. | Thank you for this helpful suggestion. We have revised the Abstract to include the rationale for using the Arksey and O’Malley (2005) framework. Specifically, we now state that this framework was selected because it provides a structured and widely used methodological approach for mapping the breadth and nature of available evidence, which is appropriate for exploring diverse interventions and policies related to access to perinatal care among PMWMIs. This clarification has been added to the Materials and Methods section of the Abstract. |
| --- | --- |
| b. Results: While the Results section highlights the quality of the interventions, it does not specify what “existing interventions and policies” were identified or how many were found. Please indicate your findings in the Results section. | Thank you for this helpful comment. We agree that the Results section needed greater clarity regarding the “existing interventions and policies” identified in the included studies. In response, we have revised the Results section to explicitly enumerate these interventions and indicate how many were found.  Specifically, we added the following paragraph at the beginning of the Results section under the thematic introduction:  “In relation to the first theme, across the 13 included studies, we identified eight existing interventions and policies designed to improve access to perinatal care for PMWMIs. These included government‑led policies such as the OHIP, the CHIP, the CHIPRA, CAWEM Plus, and state‑level public health insurance expansions, as well as non‑governmental initiatives such as MdM community‑based services. These interventions covered the full perinatal continuum, including prenatal, postpartum, and newborn‑care components such as postpartum contraceptive access and neonatal care packages. Together, these eight interventions constitute the existing policy landscape documented in the included studies.”  This revision clarifies both what the existing interventions and policies are and how many were identified, directly addressing the reviewer’s concern. |
| Introduction: a. The section would benefit from improved organization and paragraph restructuring to enhance coherence. For example, the introductory statistics presented in lines 1-8 are not sufficiently contextualized. Additionally, in lines 8- 10, it is unclear what is being contrasted, and the information on the absence of access to prenatal services appears disjointed. In line 17, kindly expand on the phrase “the restrictive measures” to clarify or highlight the potential reasons for inadequate prenatal care to strengthen the argument regarding “the growing concerns surrounding healthcare access for PMWMIS”(line 42) and “facilitate the implementation of context -appropriate intervention…” (lines 46-47). Finally, there appears to be some repetition in lines 45 to 46 and 52. | The section would benefit from improved organization and paragraph restructuring to enhance coherence. For example, the introductory statistics presented in lines 1–8 are not sufficiently contextualized. Additionally, in lines 8–10, it is unclear what is being contrasted, and the information on the absence of access to prenatal services appears disjointed.  Thank you for this valuable feedback. We have substantially revised the Introduction to improve clarity, coherence, and logical flow.  1. Contextualization of introductory statistics: We restructured the opening paragraph so that the Toronto statistics are now introduced within a broader international context. This clarifies why the figures are relevant and how they illustrate the wider issue of inadequate prenatal care among uninsured migrant women.  2. Clarification of contrasts in lines 8–10: We revised this section to clearly distinguish between insured and uninsured women, and between migrant and non‑migrant populations. The revised text now explicitly explains how insurance status influences provider type, mode of delivery, and neonatal outcomes.  3. Improved organization of information on lack of access to prenatal services: We reorganized the paragraph to ensure that the discussion of inadequate access to prenatal care follows logically from the explanation of restrictive immigration policies and insurance exclusions. This removes the previous disjointedness and strengthens the causal narrative.  These revisions enhance the coherence of the Introduction and ensure that the argument unfolds in a clear and logically structured manner. |
| Materials and Methods: a. Line 89: The phrase “newborn’s first months” is ambiguous. Please define the exact age range to improve clarity.  b. Lines 89 to 90: There appears to be a repetition of information that could be streamlined. | Thank you for this helpful observation. We have revised the text to remove ambiguity by specifying the exact age ranges covered. The phrase “newborn’s first months” has been replaced with a precise definition of the perinatal period, which now clearly states that postpartum care extends up to 42 days after delivery and newborn care extends up to 28 days of life. This revision improves clarity and aligns the terminology with established clinical definitions.  It is now read as: “This review examines care for uninsured migrants during the perinatal period and the policies that facilitate access. For the purposes of this review, the perinatal period is defined as extending from the onset of pregnancy to the early months of life, thereby expanding beyond the World Health Organization definition (29). It encompasses pregnancy testing; prenatal care (antenatal follow-up); immediate obstetric and neonatal care (during labor, delivery, and within two hours postpartum); postpartum care (up to 42 days after delivery); and newborn care (up to 28 days of life)” |
| c. Lines 95 to 97. Please provide a justification for the design inclusion and exclusion criteria. | Thank you for this helpful comment. We have now added a clear justification for our inclusion and exclusion criteria. All empirical study designs were included because the objective of this scoping review is to comprehensively map the range of interventions and policies that support perinatal care for uninsured migrant women. Given the limited and heterogeneous nature of the existing evidence, restricting the review to specific study designs would risk overlooking relevant interventions. Conversely, media articles, editorials, and studies focusing solely on the sociodemographic profiles of PMWMIs were excluded because they do not provide empirical data on interventions or policy measures, and therefore cannot contribute to answering our research question. This clarification has been added to strengthen the methodological transparency of the review. |
| d. Additionally, consider reporting the number of articles initially identified and selected to improve transparency of the review process. | Thank you for this suggestion. We have now reported the number of records identified, duplicates removed, and studies screened and included. These figures are presented in the Results section and illustrated in the PRISMA‑ScR flow diagram to enhance transparency of the review process.  The excerpt is now read as: “We identified 11,114 records and removed 4,106 duplicates. After screening titles and abstracts, an additional 7,303 records were excluded (see Flowchart). A total of 13 studies met the inclusion criteria and were included in this review (Table 1). These studies were published between 2000 and 2020.” |
| Discussion: a. Although the acronym PMWMI is defined at first use (Line 36), the Discussion section, lines 252 to 254, appears to refer to a different study population. Additionally, it is unclear whether the acronym PMWMI applies to pregnant migrant women without medical insurance (line 360) or to pregnant and postpartum women with mental Illnesses ( Line 254). Please clarify and ensure the consistent use of acronyms and study population terminology throughout the manuscript. | Thank you for pointing this out. It is a typo. The acronym **PMWMIs** is intended to refer exclusively to *pregnant migrant women without medical insurance*. The inconsistent use identified has been corrected to ensure accurate and consistent terminology throughout the manuscript. |
| b. Lines 302 to 304: Is this phrase supported by verified data? If so, please provide appropriate references. | Thank you for this comment. We have revised the sentence to ensure it is fully supported by verified data and have added appropriate references. Specifically, recent increases in migrant arrivals to the United States are now supported by Butcher and Garcia‑Jimeno (2025), who provide empirical analysis of recent immigration inflows. Additionally, the Lampedusa tragedy is now referenced using peer‑reviewed and institutional sources that document the event and its broader implications for migration pressures in the Mediterranean region (Cuttitta, 2014; Cusumano, 2019; United Nations High Commissioner for Refugees, 2013). These references substantiate the contextual examples used to illustrate how sudden population movements can place pressure on healthcare systems.  The revised text now reads: “Broader contextual examples, such as recent migration trends -including increased arrivals in the United States (70) and events such as the Lampedusa tragedy (71-73) further illustrate how sudden population movements can place pressure on healthcare systems, although these examples extend beyond the included studies.” |
| General comment: There are concerns regarding punctuation, spelling, repetition, and incomplete sentences. Please carefully reread the manuscript and make the necessary corrections. A few examples have been highlighted: Line 349: Implantation? Line 17: Per se? Line 57: incomplete | Thank you for this important observation. We have conducted a thorough review of the manuscript to correct punctuation, spelling, repetition, and sentence structure issues throughout the text. The specific examples you highlighted have been addressed as follows:   - Line 349 (“Implantation?”): The term has been revised. - Line 17 (“Per se?”): The expression has been replaced with a more precise formulation to improve readability and avoid unnecessary Latin terminology. - Line 57 (incomplete sentence): The sentence has been rewritten.   In addition to these corrections, we carefully re‑edited the entire manuscript to ensure consistency, clarity, and adherence to academic writing standards. |
