# Supplementary material for: Interventions facilitating access to perinatal care for migrant women without medical insurance: a scoping review
Source: PLoS One. 2026 Sep 10;21(9):e0357487. doi: 10.1371/journal.pone.0357487 (PMC13561353; doi:10.1371/journal.pone.0357487)
Supplement: Peer review history

# Peer review: Decision Letter 0

**DOI:** 10.1371/journal.pone.0357487.r001

**Authors:** Noushin Arefadib

2 Feb 2026

Dear Dr. Sia,

Thank you for submitting your manuscript to PLOS ONE. After careful consideration, we feel that it has merit but does not fully meet PLOS ONE’s publication criteria as it currently stands. Therefore, we invite you to submit a revised version of the manuscript that addresses the points raised during the review process.

Please submit your revised manuscript by Mar 19 2026 11:59PM. If you will need more time than this to complete your revisions, please reply to this message or contact the journal office at plosone@plos.org. When you're ready to submit your revision, log on to https://www.editorialmanager.com/pone/ and select the ‘Submissions Needing Revision’ folder to locate your manuscript file.

- A letter that responds to each point raised by the academic editor and reviewer(s). You should upload this letter as a separate file labeled ‘Response to Reviewers’.
- A marked-up copy of your manuscript that highlights changes made to the original version. You should upload this as a separate file labeled ‘Revised Manuscript with Track Changes’.
- An unmarked version of your revised paper without tracked changes. You should upload this as a separate file labeled ‘Manuscript’.

If you would like to make changes to your financial disclosure, please include your updated statement in your cover letter. Guidelines for resubmitting your figure files are available below the reviewer comments at the end of this letter.

If applicable, we recommend that you deposit your laboratory protocols in protocols.io to enhance the reproducibility of your results. Protocols.io assigns your protocol its own identifier (DOI) so that it can be cited independently in the future. For instructions see: https://journals.plos.org/plosone/s/submission-guidelines#loc-laboratory-protocols. Additionally, PLOS ONE offers an option for publishing peer-reviewed Lab Protocol articles, which describe protocols hosted on protocols.io. Read more information on sharing protocols at https://plos.org/protocols?utm_medium=editorial-email&utm_source=authorletters&utm_campaign=protocols.

We look forward to receiving your revised manuscript.

Kind regards,

Noushin Arefadib

Academic Editor

PLOS One

Journal Requirements:

When submitting your revision, we need you to address these additional requirements.

1.Please ensure that your manuscript meets PLOS ONE's style requirements, including those for file naming. The PLOS ONE style templates can be found at

https://journals.plos.org/plosone/s/file?id=wjVg/PLOSOne_formatting_sample_main_body.pdf and

https://journals.plos.org/plosone/s/file?id=ba62/PLOSOne_formatting_sample_title_authors_affiliations.pdf

2. Please note that funding information should not appear in any section or other areas of your manuscript. We will only publish funding information present in the Funding Statement section of the online submission form. Please remove any funding-related text from the manuscript.

3. Thank you for stating the following financial disclosure:

“SHERPA University Institute and the Social Sciences and Humanities Research Council (SSHRC) have funded this project”

Please state what role the funders took in the study.  If the funders had no role, please state: "The funders had no role in study design, data collection and analysis, decision to publish, or preparation of the manuscript."

If this statement is not correct you must amend it as needed.

Please include this amended Role of Funder statement in your cover letter; we will change the online submission form on your behalf.

4. Please provide a complete Data Availability Statement in the submission form, ensuring you include all necessary access information or a reason for why you are unable to make your data freely accessible. If your research concerns only data provided within your submission, please write "All data are in the manuscript and/or supporting information files" as your Data Availability Statement.

5. Please include a copy of Table 3 which you refer to in your text on page 8.

6. If the reviewer comments include a recommendation to cite specific previously published works, please review and evaluate these publications to determine whether they are relevant and should be cited. There is no requirement to cite these works unless the editor has indicated otherwise.

Additional Editor Comments:

Thank you for submitting your manuscript. The topic is timely and important; however, the manuscript requires substantial revision before it can be considered further. In addition to addressing the external reviewer comments, please carefully consider and respond to the points outlined below. These relate primarily to clarity, internal consistency, methodological rigor, and alignment between the aims, methods, results, and discussion.

Intro

- Please provide references for assertions made throughout. For example: “Four out of five uninsured pregnant women received inadequate prenatal care. More than half of these women received insufficient prenatal care, and 6.5% received no prenatal care at all.” – What are the references for these?

- “Insurance status was also linked to the type of health care provider, reasons for caesarean section, neonatal resuscitation rates, and the length of maternal hospital stay.” Was linked in what study? In what country? More transparent information required here.

- The argument in the intro is also confusing as contradictory in parts. For example, you assert: “…many migrant women in Canada and the United States often receive care that falls below recommended standards due to a lack of public health insurance coverage (6)”, but then immediately after (lines 11-15) you note that in Canada most immigrants and resettled refugees do have universal coverage comparable to citizens. Without qualification, those two claims directly clash.

- The population of interest is not clearly defined and appears inconsistent across the title and background. While the title and text refer broadly to “migrant women,” the background indicates that immigrants and resettled refugees in Canada have universal health coverage, whereas lack of insurance applies specifically to undocumented migrants and certain temporary visa holders. These groups are not interchangeable.

- The authors should clearly specify which migrant subgroups are the focus of the review, ensure alignment between the title, aims, and background, and avoid using “migrant women” as a blanket term if the focus is on uninsured subpopulations.

- There is also inconsistency in the use of “perinatal care.” While the review claims to examine perinatal interventions, the background and epidemiological data presented relate almost exclusively to antenatal care. If postnatal and intrapartum care are within scope, this should be reflected in the background and rationale. Otherwise, terminology should be tightened and used consistently from the outset.

Materials and Methods

- The sentence “This study utilized the Arksey and O’Malley” is incomplete as written and should be revised for clarity.

- The research questions and overarching aim should be presented at the end of the Introduction, not in the Methods section.

- The research questions require clarification. Research question 2 combines multiple distinct elements (impacts, strengths, weaknesses, and costs), which reduces conceptual clarity. The authors should consider streamlining or separating this question. In addition, “policies” should be clearly positioned within the scope of the review, for example as a type of intervention, and the terminology used consistently.

- For the inclusion criteria, please clarify whether a cut-off period was applied for the postpartum period.

- The inclusion criteria are unclear and partially redundant. The perinatal period is already defined by pregnancy, labour, and postpartum; the subsequent sentence referring again to “articles addressing the perinatal period” adds no additional clarification and creates ambiguity. Please streamline and clearly define the perinatal timeframe used for study inclusion.

- Lines 75-85 under “Population”: This material is conceptual and definitional and would be more appropriately located in the Introduction. Clarification of who is encompassed by the term “migrant” (and which subgroups are within scope) is necessary for framing the review, rather than being embedded in the Methods section. The population of interest should be clearly defined upfront and aligned with the title and aims.

- The content presented under “Interventions” primarily describes the population and perinatal timeframe rather than defining the intervention concept. This section should clearly specify what was considered an intervention (including policy-level interventions), what was excluded, and the rationale for these decisions. Population and timeframe definitions should be addressed in the intro and briefly in “Population” and kept distinct from intervention eligibility.

- In PICO, the comparator would be another intervention, usual care, or explicitly “no comparator”. The existing content presented under “Comparator” is not appropriate. This section describes study design, language, and publication eligibility rather than a comparator.

- The Outcomes section is incorrectly framed. Rather than describing what the review reported, this section should specify the outcome domains considered for study inclusion. So, studies were included if..

- The search strategy requires clarification. The section refers to “eleven electronic databases” but then lists journals, reference lists, and organisational websites, which are not databases. In addition, the inclusion of NGO and organisational websites appears inconsistent with the stated eligibility criteria limiting inclusion to empirical scientific studies, unless reports or grey literature were also included. The authors should clearly distinguish between databases and other sources, clarify what types of records were eligible, and ensure consistency with the stated inclusion criteria.

- Importantly: The authors note that the search was last updated in December 2024. Given that more than 12 months have elapsed, the search should be updated to ensure the review reflects the current evidence base.

- The PICO elements should be presented within the Study selection section, rather than preceding it.

- The data analysis section lacks methodological clarity. The authors describe general activities (e.g. categorising studies, summarising findings) rather than specifying the analytic approach used. Please clarify the analytical method applied (e.g. descriptive mapping, thematic synthesis, framework-based analysis), including how data were compared and synthesised.

Results

- The Results section should begin with a clear descriptive numerical summary of the included studies (e.g. total number of studies, countries, study designs, populations), consistent with scoping review reporting standards. Currently, the section opens with an interpretive statement rather than a structured overview.

- The rationale for the organisation of results under the current headings (e.g. “Interventions in favor of access…”, “Actors involved…”) is unclear. The authors should explicitly state the basis for this structure (e.g. analytic framework, thematic synthesis, a priori categories) and ensure it aligns with the described data analysis approach.

- Overall, the Results section lacks coherence and a clear analytic logic. The basis for organising results under the current headings is not specified and does not clearly align with a stated analytical framework or method. It is unclear whether findings are being grouped by intervention type, policy level, actor, outcome, or study focus, and the structure appears to shift across sections without justification.

- the section titled “Strengths and weaknesses of these interventions” is unclear. It is not specified whether this refers to the strengths and weaknesses of the interventions themselves, the policies, or the quality of the included studies. As presented, this section conflates empirical findings with interpretation and lacks methodological clarity.

- The authors should:

– Clearly state the analytic approach used to structure and synthesise results;

– Ensure that section headings are conceptually precise and consistently applied;

– Distinguish clearly between results, interpretation, and discussion-level critique.

Discussion

- The Discussion section requires tighter alignment with the aims, research questions, and findings of the review. Several sections (e.g. ethical implications, economic theory, policy agility) introduce themes that were not clearly framed in the Introduction nor explicitly examined in the Results. As written, it is often unclear whether these points are derived from the included studies or represent broader commentary.

- The authors should refocus the Discussion on interpreting the review’s findings, clearly link all discussion points to the included evidence, and avoid introducing new conceptual themes that fall outside the stated scope of the review.

[Note: HTML markup is below. Please do not edit.]

Reviewers’ comments:

Reviewer's Responses to Questions

**Comments to the Author**

1. Is the manuscript technically sound, and do the data support the conclusions?

Reviewer #1: Yes

Reviewer #2: Partly

Reviewer #3: Yes

Reviewer #4: Yes

**********

2. Has the statistical analysis been performed appropriately and rigorously?

Reviewer #1: Yes

Reviewer #2: N/A

Reviewer #3: Yes

Reviewer #4: N/A

**********

3. Have the authors made all data underlying the findings in their manuscript fully available?

The PLOS Data policy

Reviewer #1: Yes

Reviewer #2: Yes

Reviewer #3: Yes

Reviewer #4: Yes

**********

4. Is the manuscript presented in an intelligible fashion and written in standard English?

Reviewer #1: Yes

Reviewer #2: Yes

Reviewer #3: Yes

Reviewer #4: No

**********

Reviewer #1: The manuscript presents a comprehensive scoping review examining interventions and policies that influence access to perinatal care for pregnant migrant women without medical insurance (PMWMIs). It follows a clearly structured methodology guided by the PICO framework and adheres to PRISMA-ScR standards, which strengthens its methodological transparency. The inclusion and exclusion criteria are well-articulated, and the authors demonstrate thoroughness in defining “migrant” and related population subgroups, acknowledging heterogeneity across migrant statuses.

For the strengths, the use of multiple databases, grey literature, NGO websites, and hand-searching increases the review’s breadth. The involvement of an experienced librarian adds rigor to the search strategy. Furthermore, the selection process is clear with a well-structured data extraction strategy.

However, some aspects need improvement:

- Lines two to five should have a reference

- From line 11 to 31 is such a long paragraph, can be made two paragraphs

- The review is heavily dominated by studies from the USA and Canada (85%), limiting the generalizability to global migrant contexts especially low and middle-income countries where migration dynamics differ. Hence, they authors could indicate interpretation with cognisance of this

- The acronym PMWMIs is used for “pregnant migrant women without medical insurance,” but later appears to refer to postpartum women with mental illness on line 254—this should be corrected for consistency.

Reviewer #2: Thank you. This was an interesting read. The work aligned with global public health priorities, and was quite timely. This reviewer only has some minor comments.

1. Limiting to English and French would, undoubtedly, limit generalizability. There would need to be a stronger justification, and preferably a sensitivity analysis.

2. With a rather limited number of existing evidence, the authors could also consider expanind to the grey literature.

3. What was the screening process? Was there a reliability count? How exactly was the "discussion" done - the conflicts may best be quantified.

4. Was there any quality appraisal for the included studies?

5. Was it possible to deepend the synthesis and analysis? Like a thematic analysis?

6. The consultation process was mentioned, but more details should be reported.

7. Uncritically presented cost-benefit claims may not be the best way forward. Please consider applying more critial discussions, like inflation adjustments or generalizability.

8. There seemed to be some minor formatting and typo issues.

9. "First of its kind" was rather a strong claim. Please consider citing prior reviews on migrant maternal health and differentiate.

10. Please discuss more on implications on local and global policies.

Reviewer #3: This manuscript presents a well-conducted and timely scoping review addressing a critically important and underexplored area of maternal and public health: access to perinatal care for migrant women without medical insurance. The topic is highly relevant in the context of increasing global migration and persistent health inequities affecting undocumented migrants and asylum seekers.

The authors have applied the Arksey and O’Malley framework appropriately and transparently, ensuring methodological rigor throughout the scoping review process. The inclusion of both peer-reviewed literature and grey literature strengthens the comprehensiveness of the findings and enhances the policy relevance of the study. The clear description of search strategies, inclusion criteria, and study selection adds to the credibility and reproducibility of the review.

The synthesis of evidence across multiple national contexts (Canada, the United States, Iran, and Thailand) is a notable strength, as it allows for meaningful cross-country comparisons and highlights how different health system structures influence access to perinatal care. The discussion effectively links policy changes to their short-, medium-, and long-term economic and health consequences, offering valuable insights into how restrictive policies may inadvertently increase healthcare costs and worsen health outcomes.

The manuscript is clearly written, logically structured, and accessible to a broad readership, including researchers, clinicians, and policymakers. The conclusions are well supported by the presented evidence and successfully underscore the public health and ethical implications of excluding migrant women from perinatal care. Importantly, the authors’ emphasis on political drivers of policy decisions adds depth to the analysis and contributes to ongoing discussions around health equity and social justice.

Reviewer #4: Dear Authors,

Abstract:

a. Materials and Methods: Kindly include the rationale for using the Arksey and O’Malley (2005) framework in the Abstract.

b. Results: While the Results section highlights the quality of the interventions, it does not specify what “existing interventions and policies” were identified or how many were found. Please indicate your findings in the Results section.

Introduction:

a. The section would benefit from improved organization and paragraph restructuring to enhance coherence. For example, the introductory statistics presented in lines 1-8 are not sufficiently contextualized. Additionally, in lines 8- 10, it is unclear what is being contrasted, and the information on the absence of access to prenatal services appears disjointed. In line 17, kindly expand on the phrase “the restrictive measures” to clarify or highlight the potential reasons for inadequate prenatal care to strengthen the argument regarding “the growing concerns surrounding healthcare access for PMWMIS”(line 42) and “facilitate the implementation of context -appropriate intervention…” (lines 46-47). Finally, there appears to be some repetition in lines 45 to 46 and 52.

Materials and Methods:

a. Line 89: The phrase “newborn’s first months” is ambiguous. Please define the exact age range to improve clarity.

b. Lines 89 to 90: There appears to be a repetition of information that could be streamlined.

c. Lines 95 to 97. Please provide a justification for the design inclusion and exclusion criteria.

d. Additionally, consider reporting the number of articles initially identified and selected to improve transparency of the review process.

Discussion:

a. Although the acronym PMWMI is defined at first use (Line 36), the Discussion section, lines 252 to 254, appears to refer to a different study population. Additionally, it is unclear whether the acronym PMWMI applies to pregnant migrant women without medical insurance (line 360) or to pregnant and postpartum women with mental Illnesses( Line 254). Please clarify and ensure the consistent use of acronyms and study population terminology throughout the manuscript.

b. Lines 302 to 304: Is this phrase supported by verified data? If so, please provide appropriate references.

General comment: There are concerns regarding punctuation, spelling, repetition, and incomplete sentences. Please carefully reread the manuscript and make the necessary corrections. A few examples have been highlighted:

Line 349: Implantation?

Line 17: Per se?

Line 57: incomplete

**********

what does this mean?). If published, this will include your full peer review and any attached files.

If you choose “no”, your identity will remain anonymous but your review may still be made public.

**Do you want your identity to be public for this peer review?**  For information about this choice, including consent withdrawal, please see our Privacy Policy

Reviewer #1: No

Reviewer #2: No

Reviewer #3: **Yes:** Dr. Neha Asif

Reviewer #4: No

**********

[NOTE: If reviewer comments were submitted as an attachment file, they will be attached to this email and accessible via the submission site. Please log into your account, locate the manuscript record, and check for the action link "View Attachments". If this link does not appear, there are no attachment files.]

To ensure your figures meet our technical requirements, please review our figure guidelines: https://journals.plos.org/plosone/s/figures

You may also use PLOS’s free figure tool, NAAS, to help you prepare publication quality figures: https://journals.plos.org/plosone/s/figures#loc-tools-for-figure-preparation.

NAAS will assess whether your figures meet our technical requirements by comparing each figure against our figure specifications.

# Author comment: Author response to Decision Letter 1

**DOI:** 10.1371/journal.pone.0357487.r002

18 May 2026

We sincerely thank you and the reviewers for the thorough and constructive comments provided during the review process. These comments were extremely valuable in improving the clarity, methodological rigor, and overall quality of our manuscript. After revision, we believe that our manuscript has become substantially stronger.

In this revised version, we carefully addressed all comments from the Academic Editor and reviewers. Given the detailed nature and length of our responses, we provided a comprehensive point-by-point response table, which has been uploaded separately through the journal submission platform. Major revisions include:

clarification and refinement of the population of interest and terminology used throughout the manuscript;

improved alignment between the title, aims, background, methods, results, and discussion sections;

clarification of the methodological framework, research questions, eligibility criteria, and analytical approach;

restructuring and strengthening of the Results and Discussion sections to improve coherence and consistency with the review objectives;

updating the literature search to ensure inclusion of the most recent evidence;

addition and revision of tables, including the inclusion of Table 3 referred to in the manuscript;

correction of formatting, typographical, and language issues throughout the manuscript.

Attachment Submitted filename: Response to Reviewers_17_mai2026.docx

# Peer review: Decision Letter 1

**DOI:** 10.1371/journal.pone.0357487.r003

**Authors:** Sharada P Wasti

18 Aug 2026

Interventions facilitating access to perinatal care for migrant women without medical insurance: a scoping review

PONE-D-25-57301R1

Dear Drissa Sia,

Thank you all for your hard work and commitment during the revision process. Your efforts have successfully addressed all concerns and suggestions raised by the reviewers, and I am pleased to inform you that the manuscript has now been accepted for publication once it meets all outstanding technical requirements. This is an excellent outcome, and I truly appreciate the collective effort that has brought us to this stage. I look forward to the next steps in the production process and to seeing the final version published.

Within one week, you’ll receive an e-mail detailing the required amendments. When these have been addressed, you’ll receive a formal acceptance letter and your manuscript will be scheduled for publication.

An invoice will be generated when your article is formally accepted. Please note, if your institution has a publishing partnership with PLOS and your article meets the relevant criteria, all or part of your publication costs will be covered. Please make sure your user information is up-to-date by logging into Editorial Manager at Editorial Manager® and clicking the ‘Update My Information’ link at the top of the page. For questions related to billing, please contact billing support.

If your institution or institutions have a press office, please notify them about your upcoming paper to help maximize its impact. If they’ll be preparing press materials, please inform our press team as soon as possible -- no later than 48 hours after receiving the formal acceptance. Your manuscript will remain under strict press embargo until 2 pm Eastern Time on the date of publication. For more information, please contact onepress@plos.org.

Kind regards,

Sharada P Wasti, PhD

Academic Editor

PLOS One

# Editor report: Acceptance letter

**DOI:** 10.1371/journal.pone.0357487.r004

**Authors:** Sharada P Wasti

PONE-D-25-57301R1

PLOS One

Dear Dr. Sia,

I'm pleased to inform you that your manuscript has been deemed suitable for publication in PLOS One. Congratulations! Your manuscript is now being handed over to our production team.

At this stage, our production department will prepare your paper for publication. This includes ensuring the following:

* All references, tables, and figures are properly cited

* All relevant supporting information is included in the manuscript submission,

* There are no issues that prevent the paper from being properly typeset

You will receive further instructions from the production team, including instructions on how to review your proof when it is ready. Please keep in mind that we are working through a large volume of accepted articles, so please give us a few days to review your paper and let you know the next and final steps.

Lastly, if your institution or institutions have a press office, please let them know about your upcoming paper now to help maximize its impact. If they'll be preparing press materials, please inform our press team within the next 48 hours. Your manuscript will remain under strict press embargo until 2 pm Eastern Time on the date of publication. For more information, please contact onepress@plos.org.

You will receive an invoice from PLOS for your publication fee after your manuscript has reached the completed accept phase. If you receive an email requesting payment before acceptance or for any other service, this may be a phishing scheme. Learn how to identify phishing emails and protect your accounts at https://explore.plos.org/phishing.

If we can help with anything else, please email us at customercare@plos.org.

Thank you for submitting your work to PLOS One and supporting open access.

Kind regards,

PLOS One Editorial Office Staff

on behalf of

Dr. Sharada P Wasti

Academic Editor

PLOS One
